# Unique halogen–π association detected in single crystals of C–N atropisomeric *N*-(2-halophenyl)quinolin-2-one derivatives and the thione analogue

**DOI:** 10.3762/bjoc.21.138

**Published:** 2025-09-01

**Authors:** Mai Uchibori, Nanami Murate, Kanako Shima, Tatsunori Sakagami, Ko Kanehisa, Gary James Richards, Akiko Hori, Osamu Kitagawa

**Affiliations:** 1 Chemistry and Materials Program, College of Engineering, Shibaura Institute of Technology, 3-7-5 Toyosu, Kohto-ku, Tokyo 135-8548, Japan; 2 Graduate School of Engineering and Science, Shibaura Institute of Technology, 307 Fukasaku, Minuma-ku, Saitama 337-8570, Japan

**Keywords:** atropisomers, C–N bond, halogen bond, quinolinones, single crystals, thiones

## Abstract

In single crystals of C–N atropisomeric *N*-(2-halophenyl)quinolin-2-one and the thione analogue, a unique association based on a halogen–π interaction was detected. In racemic and optically pure *N*-(2-bromo- or 2-chlorophenyl)quinolin-2-ones, homochiral layered polymers, which consist of (*P*)- or (*M*)-atropisomers, were formed through intermolecular halogen–π association. The halogen–π association in the racemates is due to a halogen bond (C–X···π) between a σ-hole on the halogen atom and a π-electron on the quinolinone benzene ring, while that in optically pure forms is caused by an n–π* interaction between a lone electron pair on the halogen atom and a π* orbital of the quinolinone. In contrast to the formation of the homochiral layered polymer in quinolinones, in racemic *N*-(2-bromophenyl)quinoline-2-thione, heterochiral layered polymers, in which (*P*)- and (*M*)-atropisomers were alternately connected, were formed through an n–π* interaction between a lone electron pair on the bromine atom and a π* orbital of the quinoline-2-thione.

## Introduction

**Figure 1:**
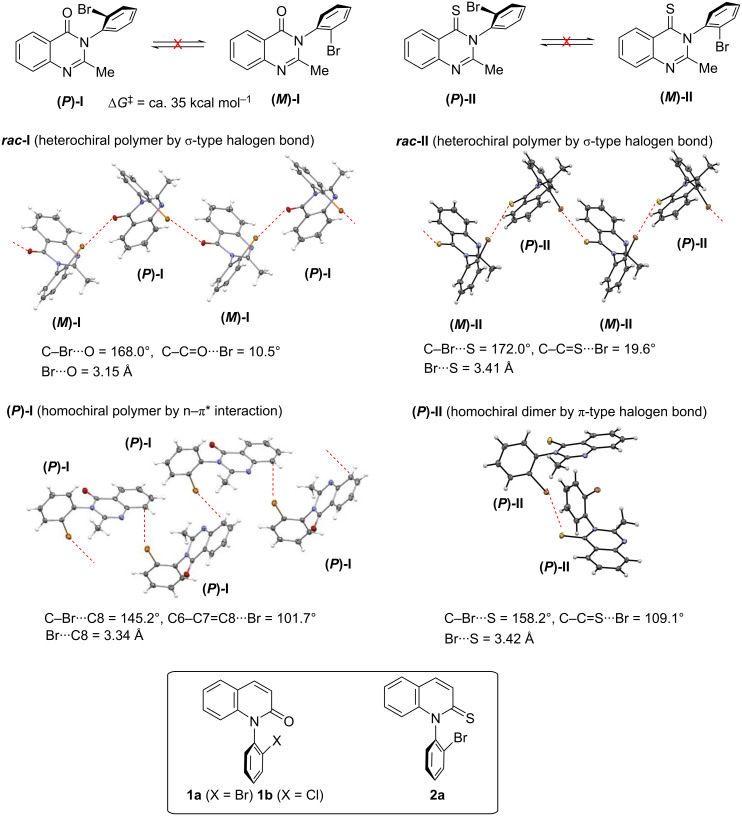
Various C–N atropisomeric compounds and their intermolecular interactions in single crystals.

In the past several years, C–N atropisomers (C–N axially chiral compounds) owing to the rotational restriction around a C–N single bond have received great attention as new target molecules for catalytic asymmetric reactions. Highly enantioselective syntheses of diverse C–N atropisomeric compounds possessing carboxamide, imide, lactam, sulfonamide, indole, pyrrole, imidazole, carbazole and amine skeletons have been reported by many groups [1–9]. C–N atropisomers are attractive compounds from the viewpoint of not only synthetic organic chemistry but also medicinal chemistry [10–13]. For example, 3-(2-bromophenyl)-2-methylquinazolin-4-one (**I**), which has a high rotational barrier about the N3–Ar bond, is known as mebroqualone possessing GABA agonist activity (Figure 1) [14–15].

Our group has been exploring asymmetric synthesis of C–N atropisomers and their structural properties for over 25 years [16–17]. As a part of the C–N atropisomeric chemistry, we succeeded in the asymmetric synthesis of mebroqualone (**I**) and the thione analogue **II** [18–19]. In the course of this study, it was found that intermolecular association in the single crystals of racemates significantly differs from that of optically pure forms (chirality-dependent halogen bonding, Figure 1) [20–21].

That is, in crystals of racemic mebroqualone ***rac*-I**, heterochiral zig-zag polymer chains, in which **(*P*)-I** and **(*M*)-I** were alternately connected, were formed through a σ-type intermolecular halogen bonding (C–Br···O) between the *ortho*-bromine atom and the carbonyl oxygen. The formation of similar heterochiral polymers through a σ-type intermolecular halogen bond (C–Br···S) was also found in crystals of the thio-analogue ***rac*-II**. On the other hand, in the optically pure mebroqualone **(*P*)-I**, the homochiral layered polymer was formed through a bromine–π association. The association is due to an n–π* interaction between a lone electron pair on the bromine atom and a π* orbital of the quinazolinone ring. In the optically pure thio-analogue **(*P*)-II**, the formation of homochiral dimers, rather than homochiral polymer chains, was detected. Furthermore, the homochiral dimers were constructed through a π-type halogen bonding (C–Br···S), rather than a σ-type such as ***rac*-I** and ***rac*-II**.

Halogen bonding has aroused great interest as a new type of noncovalent interaction as an alternative to hydrogen bonding and has been widely used as an important supramolecular tool in broad fields such as materials science, crystal engineering, liquid crystals, synthetic organic chemistry and medicinal chemistry [22–26]. Typically, halogen bonding has been classified into two major types: (1) σ-type halogen bonding, where the electrophilic region (σ-hole) of a halogen atom interacts with a lone pair on an electron-rich atom (e.g., C–X···Y), and (2) π-type halogen bonding (or halogen–π interaction), where the σ-hole interacts with an electron-rich π-system (e.g., C–X···π). The σ-hole typically forms on the extension of the C–X bond, opposite the bonding electron density, hence, the interaction is highly directional, with σ-type halogen bonds favoring nearly linear geometries (C–X···Y ≈ 180°). When the C–X···π angle significantly deviates from linearity, the halogen may act as an electron donor rather than an acceptor, and the interaction can be better described as an n–π* interaction involving the lone pair on the halogen atom.

The results shown in Figure 1 suggest that in the case of chiral compounds, the corresponding racemic and enantioenriched forms, ought to have different halogen bonding properties, and should be explored as different chemical entities. Meanwhile, there are very few studies on halogen bonding related to molecular chirality such as those shown in Figure 1 [27–30]. In addition, the studies on the comparison of intermolecular interaction (halogen bonding) between chiral compounds possessing an amide group and a thioamide group are quite rare [21].

We were curious as to whether the chirality (racemate/optically pure form)- and the functional group (C=O/C=S)-dependent halogen bonds found in **I** and **II** are also observed in other C–N atropisomeric compounds. In this article, we report the synthesis of atropisomeric *N*-(2-halophenyl)quinolin-2-ones (**1a**: X = Br, **1b**: X = Cl) and *N*-(2-bromophenyl)quinoline-2-thione (**2a**), and the analysis of halogen–mediated intermolecular interactions (halogen bonding and n–π* interaction) observed in their single crystals.

## Results and Discussion

### Synthesis of quinoline-2-ones (thione), their enantiomer separation and rotational stability

**Scheme 1:**
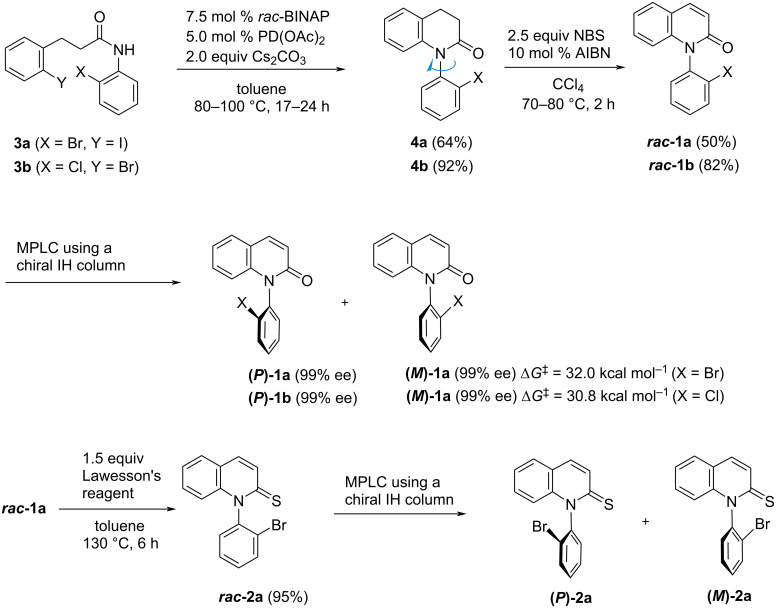
Synthesis of *N*-(2-halophenyl)quinolin-2-ones **1a**,**b** and quinoline-2-thione **2a**.

We focused on *N*-(2-halophenyl)quinolin-2-ones **1** and the thione analogue **2** as alternative substrates to verify chirality- and functional group-dependent halogen bonding observed in C–N atropisomeric quinazolinone **I** and quinazoline-thione **II**. Although the catalytic asymmetric synthesis of *N*-(2-bromo- or 2-chloro-phenyl)quinolin-2-ones **1a**,**b** was recently attempted by Doerfler et al., the yields were moderate (33% and 48%) and the enantioselectivities were poor (11% ee and 9% ee) [31]. In addition, rotational stability about the C–N bond in **1a**,**b** was not mentioned at all. We prepared racemates ***rac*-1a**,**b** in accordance with Scheme 1 and separated their enantiomers [**(*P*)-1a**,**b** and **(*M*)-1a**,**b**] through medium pressure liquid chromatography (MPLC) using a semi-preparative chiral IH column. The rotational barriers of **1a** and **1b** were evaluated to be 32.0 and 30.8 kcal mol^−1^, respectively, by a thermal racemization experiment of the separated **(*P*)-1a** and **(P)-1b**. In contrast to quinolinones **1a,b**, the atropisomers in the precursors (3,4-dihydroquinolinones **4a,b**) could not be isolated because of the low rotational stability [32].

Quinolinone ***rac*-1a** was converted to quinoline-thione ***rac*-2a** by reaction with Lawesson’s reagent, and subsequently, the enantiomers [**(*P*)-2a** and **(*M*)-2a**] were separated by MPLC using a chiral IH column. Unfortunately, the barrier value of quinoline-thione **2a** could not be evaluated because of the high rotational stability.

### Chirality dependent halogen–π interaction detected in single crystals of racemic and optically pure *N*-(2-halophenyl)quinolin-2-ones

**Figure 2:**
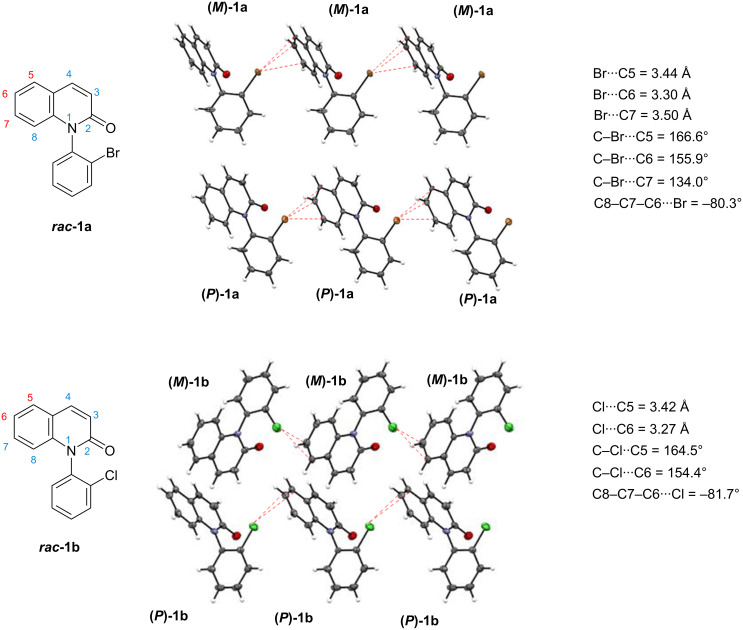
Intramolecular associations detected in crystals of ***rac*-1a** and ***rac*-1b**.

Single crystals of ***rac*-1a,b** were prepared (single crystals of ***rac*-1a,b** were obtained by vapour diffusion of hexane into a methanol solution of the compound at room temperature) and their X-ray crystal structural analyses were performed (Figure 2) [33]. In the crystals of ***rac*-1a**, a π-type intermolecular interaction between the bromine atom and the benzene ring of the quinolinone was found (the torsion angle: C8–C7–C6···Br = −80.3°). That is, the bromine atom interacts with three carbon atoms (C5–7); the three bond distances (Br···C5–7) and three bond angles (C–Br···C5–7) were 3.44, 3.30, 3.50 Å and 166.6, 155.9, 134.0°, respectively. Although a racemate, homochiral layered polymer chains, which consist of **(*P*)-1a** or **(*M*)-1a**, were formed through the Br–π interactions. The formation of homochiral layered polymers through halogen–π-type intermolecular interactions was also found in crystals of the *ortho*-chloro derivative ***rac*-1b** (the torsion angle: C8–C7–C6···Cl = −81.7°). In ***rac*-1b**, the chlorine atom interacts with two carbon atoms (C5 and C6) of the benzene ring, the two bond distances (Cl···C5,6) and two bond angles (C–Cl···C5,6) were 3.42, 3.27 Å and 164.5, 154.4°, respectively.

**Figure 3:**
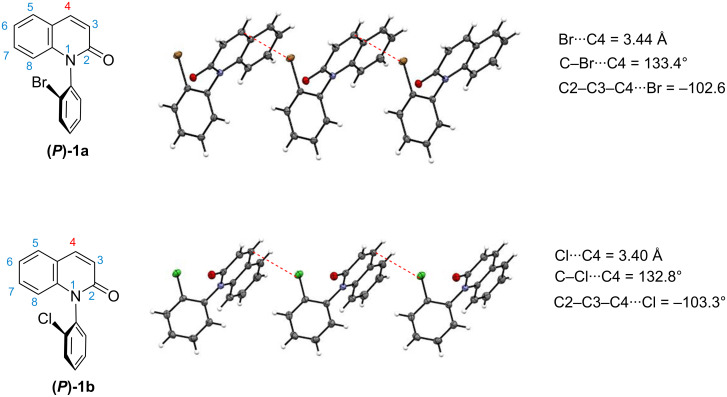
Intramolecular association detected in the crystals of **(*P*)-1a** and **(*P*)-1b**.

The formation of homochiral layered polymers through intermolecular halogen–π interactions was also observed in the crystals of optically pure forms **(*P*)-1a**,**b** (Figure 3, single crystals of **(*P*)-1a,b** were obtained by vapour diffusion of hexane into a methanol solution of the compound at room temperature) [34]. Meanwhile, the halogen atoms in **(*P*)-1a**,**b** were found to interact with the C4 atom on the lactam ring [the torsion angles of **(*P*)-1a** and **(*P*)-1b**: C2–C3–C4···Br = −102.6° and C2–C3–C4···Cl = −103.3°] but not with the benzene moiety as observed in racemates ***rac***-**1a**,**b**. The bond length (Br···C4) and bond angle (C–Br···C4) in ***(P*)-1a** were 3.44 Å and 133.4 °, respectively, and the bond length (Cl···C4) and bond angle (C–Cl···C4) in ***(P*)-1b** were 3.40 Å and 132.8 °, respectively. The C–X···C4 bonds (133.4 ° and 132.8°) in the optically pure forms **(*P*)-1a,b** were considerably bent in comparison with the C–X···C5 bonds (166.6° and 164.5°) in racemates ***rac*-1a,b**.

**Figure 4:**
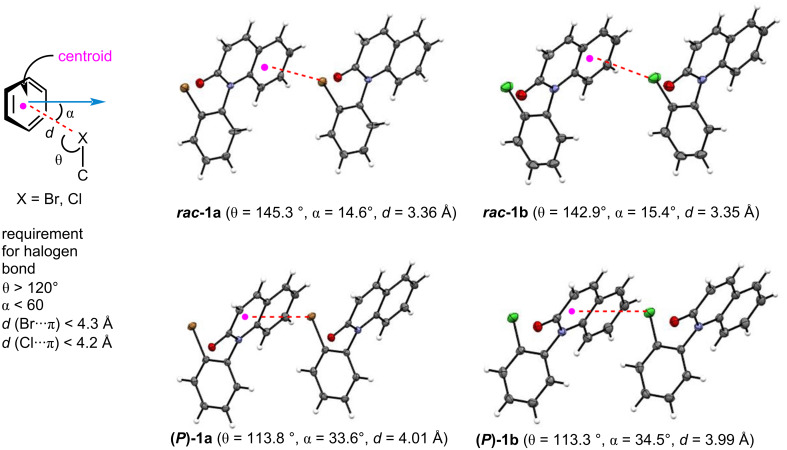
Angles (θ, α) and distances (*d*) in racemate ***rac*-1a,b** and **(*P*)-1a,b**.

Figure 4 shows the distances “*d*” and the two kinds of angles “θ”, ”α" in ***rac*-1a,b** and **(*P*)-1a,b**. The *d* values (distance between the centroid of the benzene ring and halogen X) in ***rac*-1a** and ***rac*-1b** were approximately 3.4 Å (3.36 Å and 3.35 Å, respectively), while the *d* values (distance between the centroid of the lactam ring and halogen X) in **(*P*)-1a** and **(*P*)-1b** were approximately 4.0 Å (4.01 Å and 3.99 Å, respectively). Although the *d* values in **(*P*)-1a**,**b** were 0.6 Å longer than those of ***rac*-1a**,**b**, they (4.01 Å and 3.99 Å) were within the acceptable range for halogen bonding [*d* < 4.3 Å (Br···π), *d* < 4.2 Å (Cl···π)]. The angles (θ) of C–X···(centroid) in ***rac*-1a** and ***rac*-1b** were 145.3° and 142.9°, respectively, and the angles (α) in ***rac*-1a** and ***rac*-1b** were 14.6° and 15.4°, respectively. Since both angles (θ and α) met the requirements for halogen bonding (θ > 120°, α < 60°) [33–34], the X···π interaction in ***rac*-1a**,**b** was judged to be due to halogen bonding between a σ-hole on the halogen atom and a π-electron of the aromatic ring.

On the other hand, in **(*P*)-1a** and **(*P*)-1b**, although the angles (α) were 33.6 ° and 34.5° which are acceptable for a halogen bond (α < 60°), the angles (θ) did not meet the requirement for halogen bonding (θ > 120°) [35–36]. That is, the angles (θ) in **(*P*)-1a** and **(*P*)-1b** were 113.8° and 113.3°, respectively, which are approximately 30° narrower than those in ***rac*-1a** and ***rac*-1b**. These results suggest that the C–X···π association in the optically pure forms **(*P*)-1a**,**b** is not due to halogen bonding but rather originates from an n–π* interaction between a lone electron pair on the halogen atom and a π* orbital of the quinolinone ring.

### Halogen–π interaction detected in a single crystal of racemic *N*-(2-bromophenyl)quinoline-2-thione

As mentioned in Figure 1, the intermolecular association of racemic C–N atropisomeric mebroqualone ***rac*-I** was very similar to that of the thione analogue ***rac*-II** [the formation of heterochiral polymers through a σ-type halogen bond (C–Br···O or C–Br···S)]. In contrast, the intermolecular associations in the crystals of racemic quinoline-2-thione ***rac*-2a** significantly differed from those of the racemic quinolinones ***rac*-1** in Figure 2 (single crystals of ***rac*-2a** were obtained from slow evaporation of hexane/methanol (1:1) mixture at room temperature) [37].

**Figure 5:**
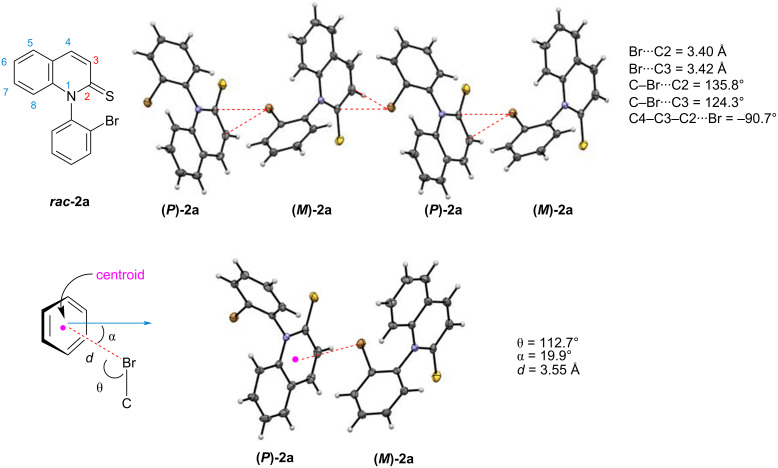
Crystal structure of racemic quinoline-2-thione ***rac*-2a**.

That is, in contrast to ***rac*-1**, in which homochiral layered polymer chains were formed, crystallization of quinoline-2-thione ***rac*-2a** led to the formation of heterochiral layered polymer chains in which **(*P*)-2a** and **(*M*)-2a** were alternately connected (Figure 5). The association between **(*P*)-2a** and **(*M*)-2a** emerged through a π-type interaction between the bromine atom and the thiolactam moiety (C4–C3–C2···Br = −90.7°). The bromine atom interacts with C2 and C3 carbons on the thiolactam ring. The *d* value of Br···(centroid of the thiolactam ring) was 3.55 Å, and the angles (θ and α) were 112.7° and 19.9°, respectively. Thus, since the angle (θ) is narrower than 120°, the Br···π association in ***rac*-2a** may be due to an n–π* interaction between a lone electron pair on the bromine atom and a π* orbital of the quinoline-thione ring, rather than a π-type halogen bonding.

Although we attempted the preparation of single crystals of the optically pure form of **2a**, unfortunately, crystals suitable for X-ray measurement could not be obtained (**2a** is chemically unstable and standing for a long period in the solution state resulted in several decomposed products).

## Conclusion

We found that crystallization of racemic and optically pure C–N atropisomeric *N*-(halophenyl)quinolin-2-one derivatives led to the formation of homochiral layered polymer chains, which consist of (*P*)- or (*M*)-atropisomers, through different types of halogen–π interactions. Homochiral layered polymers in the racemate were constructed through a π-type halogen bonding (C–X···π) between a σ-hole on the halogen atom and a π-electron of the quinoline ring, while those in the optically pure form were formed through an n–π* interaction between a lone electron pair on the halogen atom and a π*orbital of the quinolinone ring. Thus, chirality (racemic/optically pure)-dependent halogen bonding was observed in single crystals of not only 3-(2-halophenyl)quinazolin-4-one derivatives but also *N*-(2-halophenyl)quinolin-2-one derivatives. Furthermore, it was revealed that the intermolecular association of C–N atropisomeric quinoline-2-thione significantly differs from that of quinolinones. That is, in contrast to the homochiral layered polymer found in quinolinone derivatives, in the single crystal of racemic *N*-(2-bromophenyl)quinoline-2-thione, the formation of heterochiral layered polymers, in which (*P*)- and (*M*)-atropisomers were alternately connected, was detected. In addition, the heterochiral layered polymers were constructed through an n–π* interaction between the lone electron pair on the bromine atom and the π*-orbital of the quinoline-2-thione ring, rather than through π-type halogen bonding.

## Supporting Information

Crystallographic data for compounds **1** and **2** was obtained from the Cambridge Crystallographic Data Centre under deposition numbers 2448885–2448888 and 2448893. These can be obtained from the CCDC website (https://www.ccdc.cam.ac.uk/structures/).

File 1 Experimental procedures for synthesis of compounds **1**–**4** and their spectral data, copies of ^1^H and ^13^C{^1^H} NMR charts of compounds **1–4**, chiral MPLC and HPLC chart in compounds **1a**,**b**, **2a**, evaluation of rotational barriers of compounds **1a**,**b**, and X-ray crystal data of ***rac*-1a**,**b**, **(*P*)-1a**,**b**, ***rac*-2a** (check CIF).

## Data Availability

All data that supports the findings of this study is available in the published article and/or the supporting information of this article.
