# Supplementary material for: Unique halogen–π association detected in single crystals of C–N atropisomeric N-(2-halophenyl)quinolin-2-one derivatives and the thione analogue
Source: Beilstein J Org Chem. 2025 Sep 1;21:1748–56. doi: 10.3762/bjoc.21.138 (PMC12415909; doi:10.3762/bjoc.21.138)

BEILSTEIN JOURNAL OF ORGANIC CHEMISTRY

Supporting Information

for

Unique halogen–π association detected in single crystals of C–N atropisomeric *N*-(2-halophenyl)quinolin-2-one derivatives and the thione analogue

Mai Uchibori, Nanami Murate, Kanako Shima, Tatsunori Sakagami, Ko Kanehisa, Gary James Richards, Akiko Hori and Osamu Kitagawa

Beilstein J. Org. Chem. **2025,** *21,* 1748–1756. doi:10.3762/bjoc.21.138

Experimental procedures for synthesis of compounds 1–4 and their spectral data, copies of ^{1}H and $^{13}C\{^{1}H\}$ NMR charts of compounds 1–4, chiral MPLC and HPLC chart in compounds 1a,b, 2a, evaluation of rotational barriers of compounds 1a,b, and X-ray crystal data of *rac*-1a,b, (*P*)-1a,b, *rac*-2a (check CIF)

License and Terms: This is a supporting information file under the terms of the Creative Commons Attribution License (https://creativecommons.org/licenses/by/4.0). Please note that the reuse, redistribution and reproduction in particular requires that the author(s) and source are credited and that individual graphics may be subject to special legal provisions.
The license is subject to the *Beilstein Journal of Organic Chemistry* terms and conditions: (https://www.beilstein-journals.org/bjoc/terms)

Table of Contents

Experimental procedures for synthesis of compounds **1**–**4** and their spectral data S2–S4

Copies of ^{1}H and $^{13}C\{^{1}H\}$ NMR charts of compounds **1–4** S5–S11

Chiral MPLC and HPLC charts of compounds **1a**,**b** and **2a** S12–S19

Evaluation of rotational barriers of compounds **1a** and **1b** S20–S21

X-ray crystal data of ***rac*-1a,b**, **(*P*)-1a**,**b**, ***rac*-2a** (check CIF) S22–S41

EXPERIMENTAL SECTION

Experimental procedures for synthesis of compounds 1–4 and their spectral data

Melting points were recorded on a melting point apparatus and are uncorrected. ^{1}H and ^{13}C NMR spectra were recorded on 400 MHz and 101 MHz spectrometer. Chemical shifts δ were given in ppm and coupling constants *J* in Hz. ^{1}H and ^{13}C NMR chemical shifts were determined using residual signals of the deuterated solvents: $CDCl_3$ (^{1}H: δ = 7.26 ppm, ^{13}C: δ = 77.0 ppm). HRMS were recorded on a double focusing magnetic sector mass spectrometer using ESI-TOF. Optical rotations were measured in $CHCl_3$ on JASCO P-1020 Polarimeter at λ = 589 nm. $[\alpha]_D$ values were reported at 25 °C in degree·cm^2·g^{-1} with concentrations reported in g/100 mL. Column chromatography was performed on silica gel (63–210 μm). Medium-pressure liquid chromatography (MPLC) was performed on a 25 × 4 cm i. d. prepacked column (silica gel, 10 μm) with a UV detector. High-performance liquid chromatography (HPLC) was performed on a 25 × 0.4 cm i. d. chiral column with a UV detector. The single crystal X-ray structure was determined by a Bruker D8 Quest with MoK$_\alpha$ radiation (λ = 0.71073 Å) generated at 50 kV and 1 mA. The crystal was coated by paratone-N oil and measured at 100 K.

***N*-(2-Bromophenyl)-3-(2-iodophenyl)propanamide (3a)**. Under a N_2 atmosphere, to 3-(2-iodophenyl)propanoic acid (552 mg, 2.0 mmol) and 3-ethylcarbodiimide hydrochloride (575 mg, 3.0 mmol) was added 2-bromoaniline (0.23 mL, 2.1 mmol) in CH_2Cl_2 (0.8 mL) at 0 °C and then the mixture was stirred for 5 h at rt. The mixture was poured into 2 N HCl aq and extracted with AcOEt. The AcOEt extracts were washed with brine, dried over $MgSO_4$, and evaporated to dryness in vacuo. Purification of the residue by column chromatography (hexane/AcOEt = 8) gave **3a** (561 mg, 65%). **3a**: white solid; mp 150–151 °C; IR (neat) 3265, 3254, 1659 cm^{-1}; ^{1}H NMR (400 MHz, $CHCl_3$) *δ*: 8.36 (1H, d, *J* = 8.0 Hz), 7.83 (1H, dd, *J* = 8.4, 1.0 Hz), 7.62 (1H, brs), 7.51 (1H, dd, *J* = 8.0, 1.2 Hz), 7.26–7.33 (3H, m), 6.96 (1H, td, *J* = 8.0, 1.6 Hz), 6.92 (1H, m), 3.18 (2H, t, *J* = 8.0 Hz), 2.72 (2H, t, *J* = 8.0 Hz); ^{13}C{^{1}H} NMR (101 MHz, $CHCl_3$) *δ*: 169.8, 142.7, 139.6, 135.5, 132.2, 129.7, 128.6, 128.33, 128.32, 125.1, 121.9, 113.2, 100.2, 38.1, 36.6; MS (ESI-TOF) *m/z:* [MH]$^+$ 430; HRMS (ESI-TOF) *m/z*: [MH]$^+$ Calcd for $C_{15}H_{14}{}^{79}BrINO$ 429.9303; Found 429.9305.

***N*-(2-Chlorophenyl)-3-(2-bromophenyl)propanamide (3b)**. **3b** was prepared from 3-(2-bromophenyl)propanoic acid (687 mg, 3.0 mmol) and 2-chloroaniline (0.30 mL, 2.8 mmol) in accordance with the experimental procedure for the synthesis of **3a**. Purification of the residue by column chromatography (hexane/AcOEt = 11) gave **3b** (687 mg, 68%). **3b**: white solid; mp 126 °C; IR (neat) 3271, 1659 cm^{-1}; ^{1}H NMR (400 MHz, $CHCl_3$) *δ*: 8.38 (1H, d, *J* = 8.0 Hz), 7.57 (1H, brs), 7.56 (1H, dd, *J* = 8.0, 1.2 Hz), 7.22–7.36 (4H, m), 7.09 (1H, td, *J* = 8.0, 1.6 Hz), 7.03 (1H, td, *J* = 8.0, 1.6 Hz), 3.20 (2H, t, *J* = 7.8 Hz), 2.76 (2H, t, *J* = 7.8 Hz); ^{13}C{^{1}H} NMR (101

MHz, $CHCl_3$) *δ*: 170.0, 139.5, 134.4, 132.9, 130.7, 128.9, 128.2, 127.73, 127.70, 124.6, 124.3, 122.5, 121.6, 37.7, 32.0; MS (ESI-TOF) $[MH]^+$ 338; HRMS (ESI-TOF) *m/z*: $[MH]^+$ Calcd for $C_{15}H_{14}{}^{79}BrClNO$ 337.9947; Found 337.9943.

1-(2-Bromophenyl)-3,4-dihydroquinolin-2(1*H*)-one (4a). Under a N_2 atmosphere, to *rac*-BINAP (98 mg, 0.16 mmol), $Pd(OAc)_2$ (23 mg, 0.1 mmol) and Cs_2CO_3 (1.312 g, 4.0 mmol) was added toluene (10 mL), and then the mixture was stirred for 15 min at rt. **3a** (866 mg, 2.0 mmol) was added to the mixture. After being stirred for 17 h at 90 °C (oil bath), the mixture was poured into 2N HCl aq and extracted with AcOEt. The AcOEt extracts were washed with brine, dried over $MgSO_4$, and evaporated to dry-ness in vacuo. Purification of the residue by column chromatography (hexane/AcOEt = 6) gave **4a** (388 mg, 64%). ^{1}H NMR and ^{13}C NMR spectra of **4a** coincided with those described in the literature.[1]

1-(2-Chlorophenyl)-3,4-dihydroquinolin-2(1*H*)-one (4b). **4b** was prepared from **3b** (215 mg, 0.63 mmol) in accordance with the experimental procedure for the synthesis of **4a**. Purification of the residue by column chromatography (hexane/AcOEt = 3) gave **4b** (151 mg, 92%). ^{1}H NMR and ^{13}C NMR spectra of **4b** coincided with those described in the literature.[1]

1-(2-Bromophenyl)quinolin-2(1*H*)-one (*rac*-1a). Under a N_2 atmosphere, to NBS (316 mg, 1.8 mmol) and AIBN (12 mg, 0.07 mmol) was added **4a** (215 mg, 0.7 mmol) in CCl_4 (4.0 mL). The mixture was stirred for 2 h at 80 °C (oil bath). The mixture was poured into saturated $Na_2S_2O_3$ aq and extracted with AcOEt. The AcOEt extracts were washed with brine, dried over $MgSO_4$, and evaporated to dryness in vacuo. Purification of the residue by column chromatography (hexane/AcOEt = 3) gave ***rac*-1a** (106 mg, 50%). ^{1}H NMR and ^{13}C NMR spectra of ***rac*-1a** coincided with those described in the literature.[2]

1-(2-Chlorophenyl)quinolin-2(1*H*)-one (*rac*-1b). ***Rac*-1b** was prepared from **4b** (258 mg, 1.0 mmol) in accordance with the experimental procedure for the synthesis of ***rac*-1a**. Purification of the residue by column chromatography (hexane/AcOEt = 3) gave ***rac*-1b** (211 mg, 82%). ^{1}H NMR and ^{13}C NMR spectra of ***rac*-1b** coincided with those described in the literature.[2]

Enantiomer separation of *rac* -1a. Enantiomers of ***rac*-1a** were separated by MPLC using a chiral semi-preparative IH column [eluent: 15% IPA in hexane, flow rate, 4.0 mL/min; **(*P*)-1a**, t_R = 18.0 min; **(*M*)-1a**, t_R = 33.1 min]. The ee (99% ee) of **(*P*)-1a** and **(*M*)-1a** was determined by HPLC analysis using a chiral column (CHIRALPAK AD-H) [25 cm × 0.46 cm (inside diameter); 15% IPA in hexane; flow rate, 0.8 mL/min; **(*P*)-1a**, t_R = 12.7 min; **(*M*)-1a**, t_R = 20.1 min]. **(*P*)-1a**: white solid; mp 135–137 °C; $[\alpha]_D^{25}$ = + 93.9 ° (c = 0.32, $CHCl_3$). **(*M*)-1a**: $[\alpha]_D^{25}$ = –87.1 ° (c = 0.32, $CHCl_3$).

Enantiomer separation of *rac* -1b. Enantiomers of ***rac*-1b** were separated by MPLC using a chiral semi-preparative IH column [eluent: 15% IPA in hexane, flow rate, 4.0 mL/min; **(*P*)-1b**, t_R = 14.9 min; **(*M*)-1b**, t_R = 30.1 min]. The ee (99% ee) of **(*P*)-1b** and **(*M*)-1b** was determined by

HPLC analysis using a chiral column (CHIRALPAK AD-H) [25 cm × 0.46 cm (inside diameter); 15% IPA in hexane; flow rate, 0.8 mL/min; **(*P*)-1b**, t_R = 11.3 min; **(*M*)-1b**, t_R = 18.2 min]. **(*P*)-1b**: white solid; mp 158–159 °C; $[\alpha]_D^{25}$ = + 94.0 ° (c = 0.33, $CHCl_3$). **(*M*)-1b**: $[\alpha]_D^{25}$ = –82.2 ° (c = 0.33, $CHCl_3$)

1-(2-Bromophenyl)quinoline-2(1*H*)-thione (*rac*-2a). Under a N_2 atmosphere, to Lawesson's reagent (211 mg, 0.52 mmol) was added ***rac*-1a** (105 mg, 0.35 mmol) in toluene (4.5 mL). The mixture was stirred for 6 h at under refluxing toluene (oil bath 130 °C). The mixture was poured into H_2O and extracted with AcOEt. The AcOEt extracts were washed with brine, dried over $MgSO_4$, and evaporated to dryness in vacuo. Purification of the residue by column chromatography (hexane/AcOEt = 8) gave ***rac*-2a** (105 mg, 95%). ***rac*-2a**: yellow solid; mp 149–150 °C; IR (neat) 1609,1553 cm^{-1}; ^{1}H NMR (400 MHz, $CHCl_3$) δ: 7.85 (1H, dd, J = 8.0, 1.6 Hz), 7.69(1H, dd, J = 7.6, 1.6 Hz), 7.68 (1H, d, J = 9.2 Hz), 7.57–7.61 (2H, m), 7.40–7.46 (2H, m), 7.30–7.34 (2H, m), 6.61 (1H, d, J = 8.4 Hz) $^{13}C\{^1H\}$ NMR (101 MHz, $CHCl_3$) δ: 185.0, 141.1, 140.4, 134.5, 133.6, 132.9, 131.1, 130.6, 130.2, 129.5, 128.4, 124.3, 123.7, 122.4, 116.5; MS (ESI-TOF) *m/z:* $[MNa]^+$ 340; HRMS (ESI-TOF) *m/z*: $[MNa]^+$ Calcd for $C_{15}H_{10}{}^{81}BrNNaS$ 339.9595; Found 339.9601.

Enantiomer separation of *rac*-2a. Enantiomers of ***rac*-2a** were separated by MPLC using a chiral semi-preparative IH column (eluent: 5% IPA in hexane, flow rate, 4.0 mL/min; **(+)-2a**, t_R = 34.6 min; **(–)-2a**, t_R = 43.7 min]. The ee (99% ee) of **(+)-2a** and **(–)-2a** was determined by HPLC analysis using a chiral column (CHIRALPAK AS-H) [25 cm × 0.46 cm (inside diameter); 15% IPA in hexane; flow rate, 0.8 mL/min; **(+)-2a**, t_R = 20.1 min; **(–)-2a**, t_R = 26.1 min]. **(+)-2a**: amorphous solid; $[\alpha]_D^{25}$ = + 160.0 ° (c = 0.20, $CHCl_3$). **(–)-2a**: $[\alpha]_D^{25}$ = –143 ° (c = 0.20, $CHCl_3$).

(References)

1. Wu, L.; Zhou, H.; Sun, L.; Cui, J.; Liu, W.; Wang, Y.; Xie, L. Visible Light-Induced Photocatalyst-Free Aromatic Amidation for the Synthesis of 3,4-Dihydroquinolin-2(1H)-ones. *Russian J. Org. Chem.* **2024**, *60*, 459–466. https://link.springer.com/article/10.1134/S1070428024030138
2. Arunachalampillai, A.; Chandrappa, P.; Cherney, A.; Crockett, R.; Doerfler, J. Johnson, G.; Kommuri, V. C.; Kyad, A.; Macmanus, J. Murray, J. et al. Atroposelective Brønsted Acid-Catalyzed Photocyclization to Access Chiral N−Aryl Quinolones with Low Rotational Barriers. *Org. Lett.* **2023**, *25*, 5856–5861. https://doi.org/10.1021/acs.orglett.3c02117

Copies of ^{1}H and ^{13}C{^{1}H} NMR charts of compounds 1–4

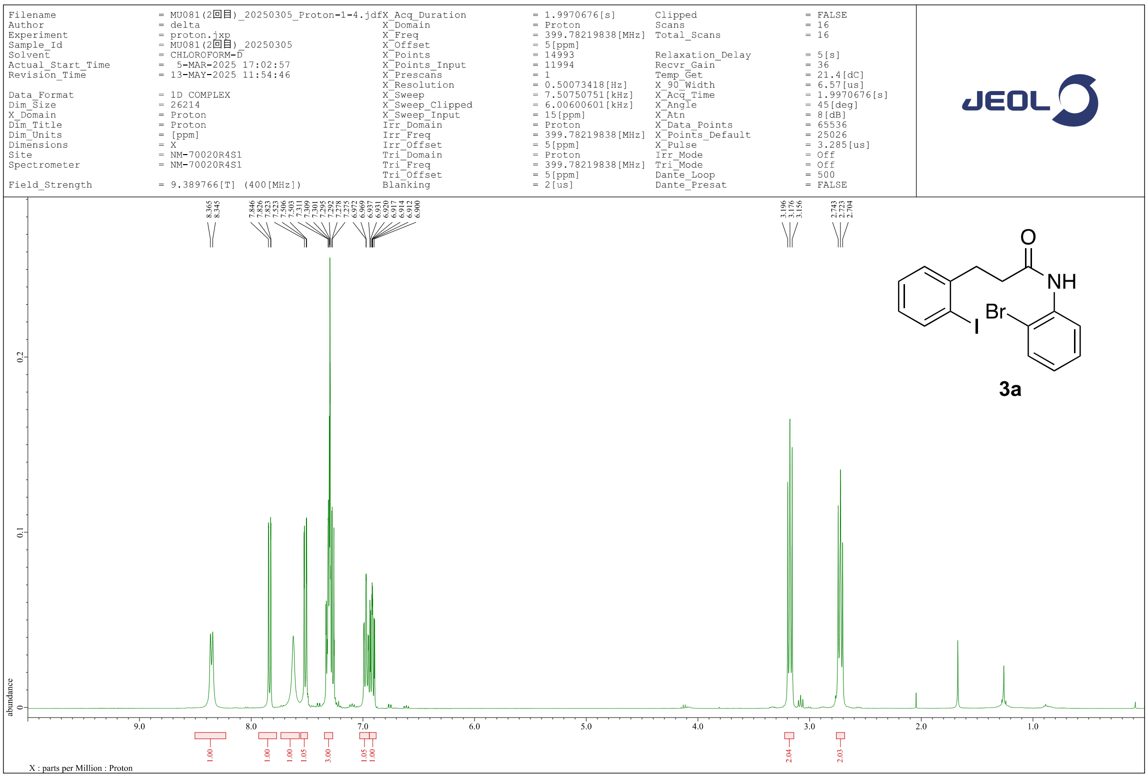

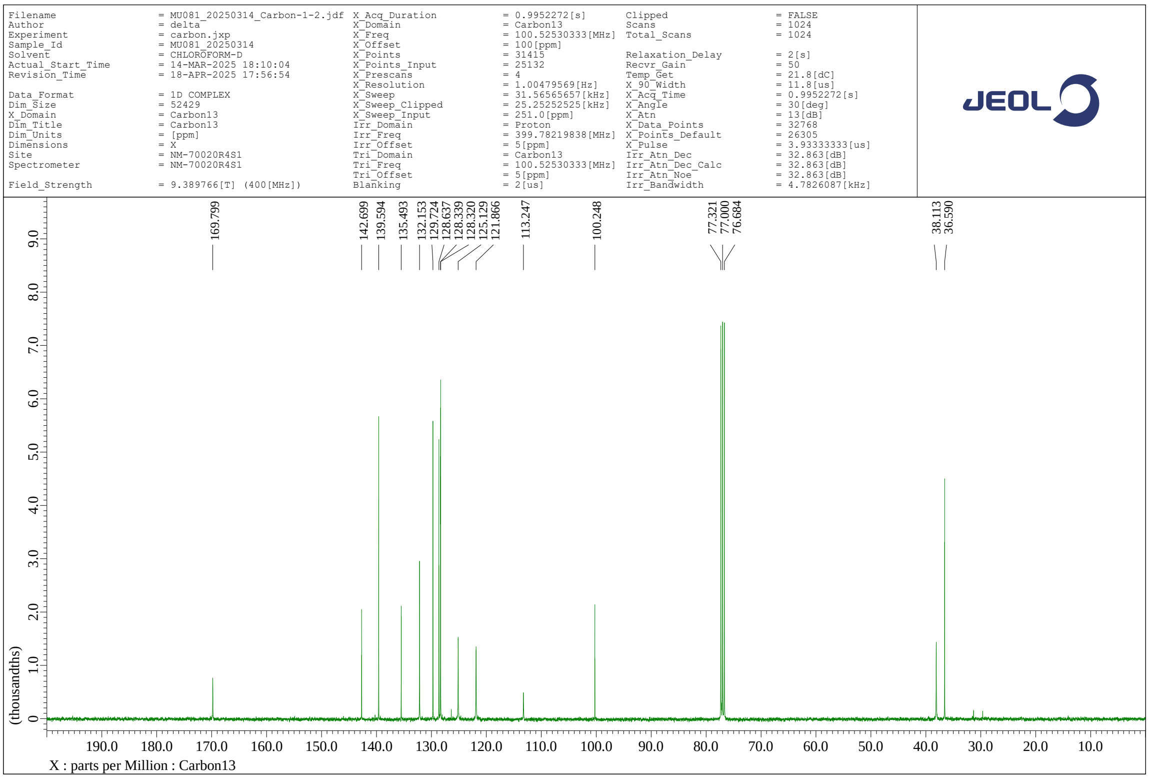

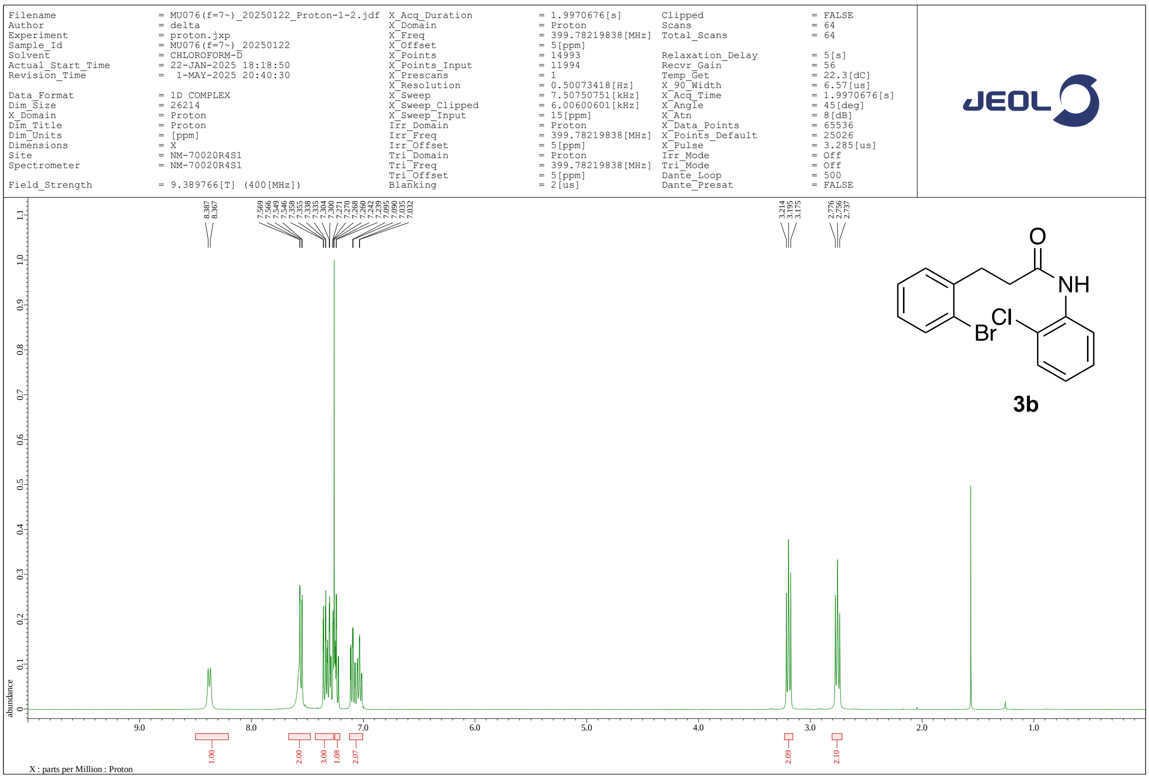
Filename = MU076(f=7~)_20250122_Proton-1-2.jdf
Author = delta
Experiment = proton.jxp
Sample_Id = MU076(f=7~)_20250122
Solvent = CHLOROFORM-D
Actual_Start_Time = 22-JAN-2025 18:18:50
Revision_Time = 1-MAY-2025 20:40:30
Data_Format = 1D COMPLEX
Dim_Size = 26214
X_Domain = Proton
Dim_Title = Proton
Dim_Units = [ppm]
Dimensions = X
Site = NM-70020R4S1
Spectrometer = NM-70020R4S1
Field_Strength = 9.389766[T] (400[MHz])
X_Acq_Duration = 1.9970676[s]
X_Domain = Proton
X_Freq = 399.78219838[MHz]
X_Offset = 5[ppm]
X_Points = 14993
X_Points_Input = 11994
X_Prescans = 1
X_Resolution = 0.50073418[Hz]
X_Sweep = 7.50750751[kHz]
X_Sweep_Clipped = 6.00600601[kHz]
X_Sweep_Input = 15[ppm]
Irr_Domain = Proton
Irr_Freq = 399.78219838[MHz]
Irr_Offset = 5[ppm]
Tri_Domain = Proton
Tri_Freq = 399.78219838[MHz]
Tri_Offset = 5[ppm]
Blanking = 2[us]
Clipped = FALSE
Scans = 64
Total_Scans = 64
Relaxation_Delay = 5[s]
Recvr_Gain = 56
Temp_Get = 22.3[dC]
X_90_Width = 6.57[us]
X_Acq_Time = 1.9970676[s]
X_Angle = 45[deg]
X_Atn = 8[dB]
X_Data_Points = 65536
X_Points_Default = 25026
X_Pulse = 3.285[us]
Irr_Mode = Off
Tri_Mode = Off
Dante_Loop = 500
Dante_Presat = FALSE
JEOL
8.387 8.367
7.569 7.566 7.549 7.546 7.358 7.355 7.338 7.335 7.304 7.300 7.271 7.270 7.268 7.260 7.242 7.239 7.095 7.090 7.035 7.032
3.214 3.195 3.175
2.776 2.756 2.737
O
NH
Cl
Br
3b
1.00 2.00 3.00 1.08 2.07 2.09 2.10
abundance
X : parts per Million : Proton

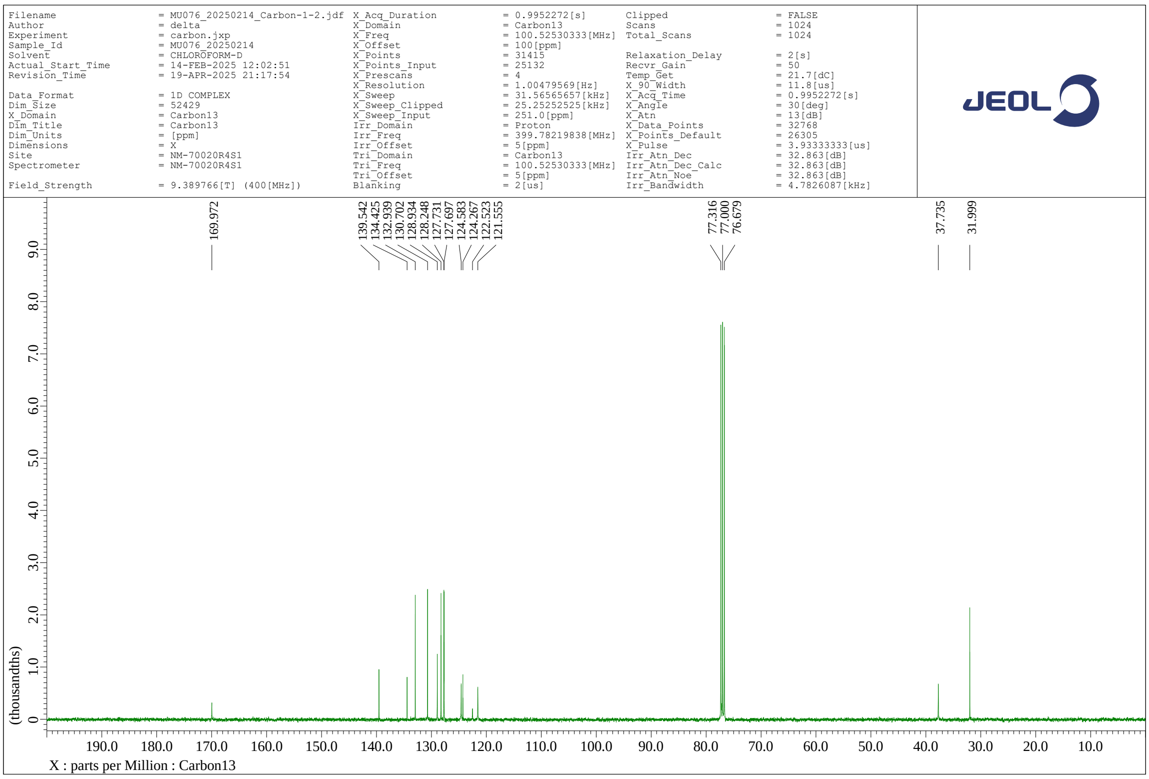
Filename = MU076_20250214_Carbon-1-2.jdf
Author = delta
Experiment = carbon.jxp
Sample_Id = MU076_20250214
Solvent = CHLOROFORM-D
Actual_Start_Time = 14-FEB-2025 12:02:51
Revision_Time = 19-APR-2025 21:17:54
Data_Format = 1D COMPLEX
Dim_Size = 52429
X_Domain = Carbon13
Dim_Title = Carbon13
Dim_Units = [ppm]
Dimensions = X
Site = NM-70020R4S1
Spectrometer = NM-70020R4S1
Field_Strength = 9.389766[T] (400[MHz])
X_Acq_Duration = 0.9952272[s]
X_Domain = Carbon13
X_Freq = 100.52530333[MHz]
X_Offset = 100[ppm]
X_Points = 31415
X_Points_Input = 25132
X_Prescans = 4
X_Resolution = 1.00479569[Hz]
X_Sweep = 31.56565657[kHz]
X_Sweep_Clipped = 25.25252525[kHz]
X_Sweep_Input = 251.0[ppm]
Irr_Domain = Proton
Irr_Freq = 399.78219838[MHz]
Irr_Offset = 5[ppm]
Tri_Domain = Carbon13
Tri_Freq = 100.52530333[MHz]
Tri_Offset = 5[ppm]
Blanking = 2[us]
Clipped = FALSE
Scans = 1024
Total_Scans = 1024
Relaxation_Delay = 2[s]
Recvr_Gain = 50
Temp_Get = 21.7[dC]
X_90_Width = 11.8[us]
X_Acq_Time = 0.9952272[s]
X_Angle = 30[deg]
X_Atn = 13[dB]
X_Data_Points = 32768
X_Points_Default = 26305
X_Pulse = 3.93333333[us]
Irr_Atn_Dec = 32.863[dB]
Irr_Atn_Dec_Calc = 32.863[dB]
Irr_Atn_Noe = 32.863[dB]
Irr_Bandwidth = 4.7826087[kHz]
JEOL
169.972
139.542 134.425 132.939 130.702 128.934 128.248 127.731 127.697 124.583 124.267 122.523 121.555
77.316 77.000 76.679
37.735
31.999
(thousandths)
X : parts per Million : Carbon13

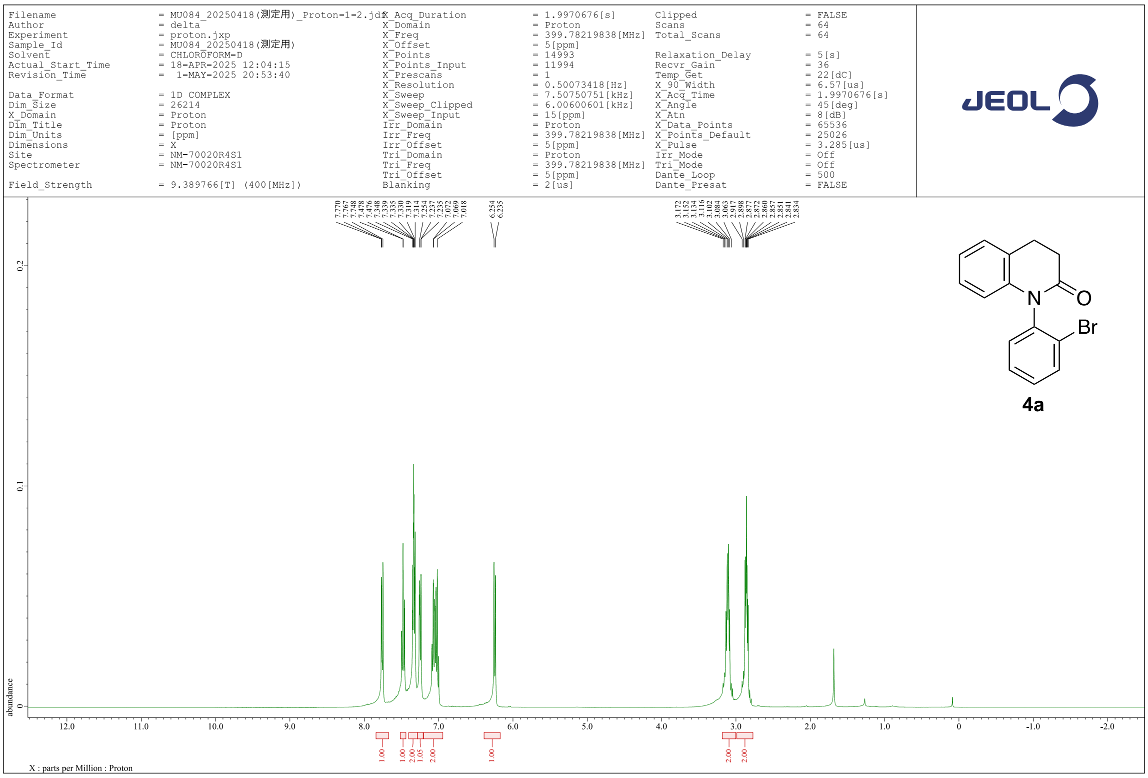
Filename = MU084_20250418(測定用)_Proton-1-2.jdf
Author = delta
Experiment = proton.jxp
Sample_Id = MU084_20250418(測定用)
Solvent = CHLOROFORM-D
Actual_Start_Time = 18-APR-2025 12:04:15
Revision_Time = 1-MAY-2025 20:53:40
Data_Format = 1D COMPLEX
Dim_Size = 26214
X_Domain = Proton
Dim_Title = Proton
Dim_Units = [ppm]
Dimensions = X
Site = NM-70020R4S1
Spectrometer = NM-70020R4S1
Field_Strength = 9.389766[T] (400[MHz])
X_Acq_Duration = 1.9970676[s]
X_Domain = Proton
X_Freq = 399.78219838[MHz]
X_Offset = 5[ppm]
X_Points = 14993
X_Points_Input = 11994
X_Prescans = 1
X_Resolution = 0.50073418[Hz]
X_Sweep = 7.50750751[kHz]
X_Sweep_Clipped = 6.00600601[kHz]
X_Sweep_Input = 15[ppm]
Irr_Domain = Proton
Irr_Freq = 399.78219838[MHz]
Irr_Offset = 5[ppm]
Tri_Domain = Proton
Tri_Freq = 399.78219838[MHz]
Tri_Offset = 5[ppm]
Blanking = 2[us]
Clipped = FALSE
Scans = 64
Total_Scans = 64
Relaxation_Delay = 5[s]
Recvr_Gain = 36
Temp_Get = 22[dC]
X_90_Width = 6.57[us]
X_Acq_Time = 1.9970676[s]
X_Angle = 45[deg]
X_Atn = 8[dB]
X_Data_Points = 65536
X_Points_Default = 25026
X_Pulse = 3.285[us]
Irr_Mode = Off
Tri_Mode = Off
Dante_Loop = 500
Dante_Presat = FALSE
JEOL
7.770 7.767 7.748 7.478 7.476 7.348 7.339 7.335 7.330 7.319 7.314 7.254 7.237 7.235 7.072 7.069 7.018
6.254 6.235
3.172 3.152 3.134 3.116 3.102 3.084 3.063 2.917 2.898 2.877 2.872 2.860 2.857 2.851 2.841 2.834
N
O
Br
4a
abundance
0 0.1 0.2
12.0 11.0 10.0 9.0 8.0 7.0 6.0 5.0 4.0 3.0 2.0 1.0 0 -1.0 -2.0
1.00 1.00 2.00 1.05 2.00 1.00 2.00 2.00
X : parts per Million : Proton

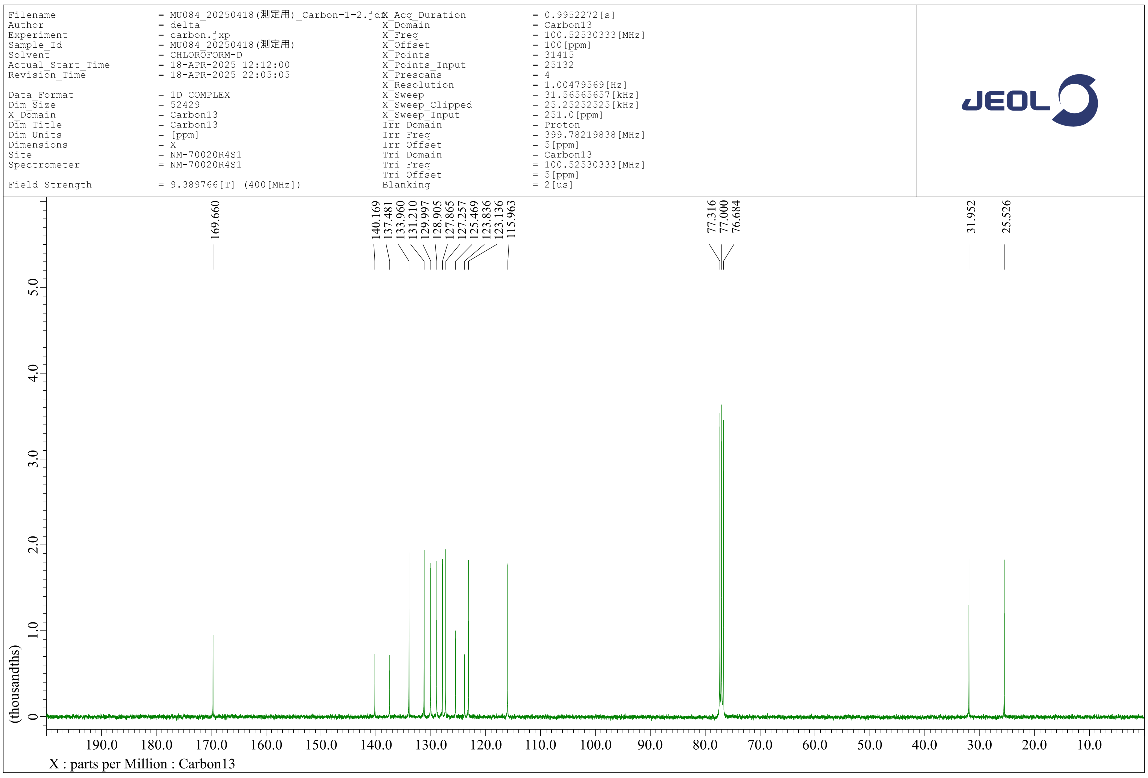
Filename = MU084_20250418(測定用)_Carbon-1-2.jdf
Author = delta
Experiment = carbon.jxp
Sample_Id = MU084_20250418(測定用)
Solvent = CHLOROFORM-D
Actual_Start_Time = 18-APR-2025 12:12:00
Revision_Time = 18-APR-2025 22:05:05
Data_Format = 1D COMPLEX
Dim_Size = 52429
X_Domain = Carbon13
Dim_Title = Carbon13
Dim_Units = [ppm]
Dimensions = X
Site = NM-70020R4S1
Spectrometer = NM-70020R4S1
Field_Strength = 9.389766[T] (400[MHz])
X_Acq_Duration = 0.9952272[s]
X_Domain = Carbon13
X_Freq = 100.52530333[MHz]
X_Offset = 100[ppm]
X_Points = 31415
X_Points_Input = 25132
X_Prescans = 4
X_Resolution = 1.00479569[Hz]
X_Sweep = 31.56565657[kHz]
X_Sweep_Clipped = 25.25252525[kHz]
X_Sweep_Input = 251.0[ppm]
Irr_Domain = Proton
Irr_Freq = 399.78219838[MHz]
Irr_Offset = 5[ppm]
Tri_Domain = Carbon13
Tri_Freq = 100.52530333[MHz]
Tri_Offset = 5[ppm]
Blanking = 2[us]
JEOL
169.660
140.169 137.481 133.960 131.210 129.997 128.905 127.865 127.257 125.469 123.836 123.136 115.963
77.316 77.000 76.684
31.952 25.526
(thousandths)
0 1.0 2.0 3.0 4.0 5.0
190.0 180.0 170.0 160.0 150.0 140.0 130.0 120.0 110.0 100.0 90.0 80.0 70.0 60.0 50.0 40.0 30.0 20.0 10.0
X : parts per Million : Carbon13

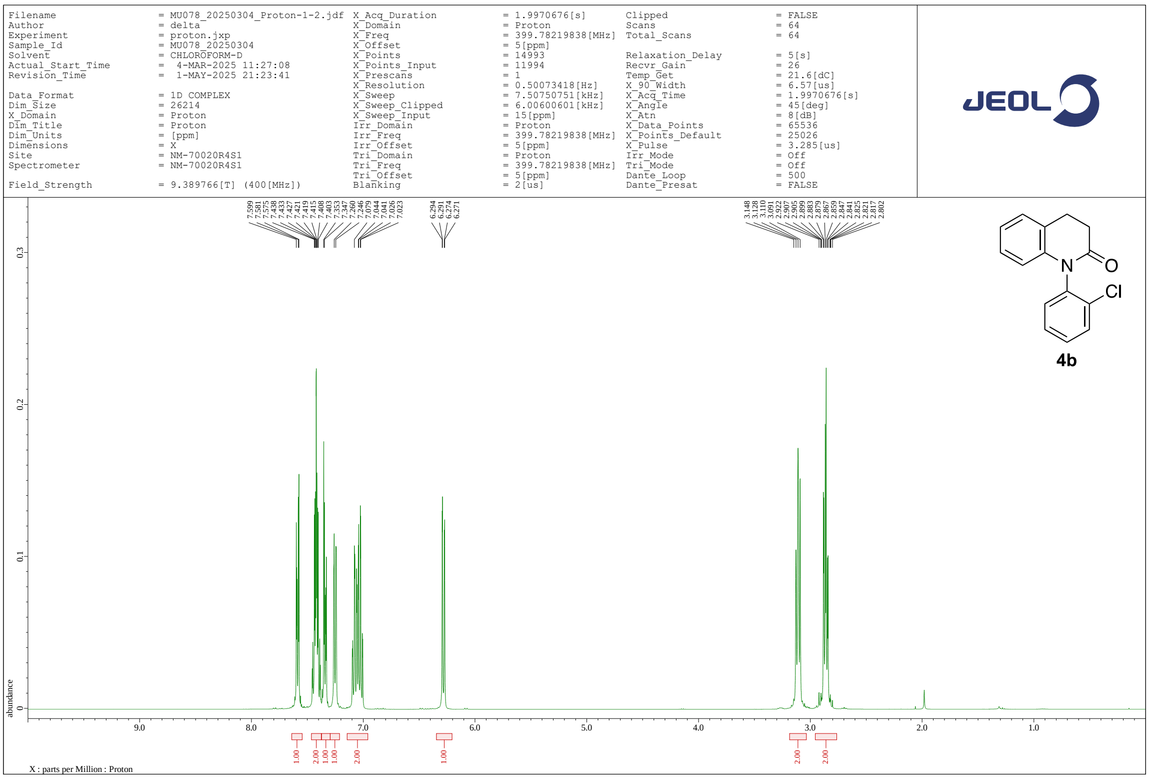
Filename = MU078_20250304_Proton-1-2.jdf
Author = delta
Experiment = proton.jxp
Sample_Id = MU078_20250304
Solvent = CHLOROFORM-D
Actual_Start_Time = 4-MAR-2025 11:27:08
Revision_Time = 1-MAY-2025 21:23:41
Data_Format = 1D COMPLEX
Dim_Size = 26214
X_Domain = Proton
Dim_Title = Proton
Dim_Units = [ppm]
Dimensions = X
Site = NM-70020R4S1
Spectrometer = NM-70020R4S1
Field_Strength = 9.389766[T] (400[MHz])
X_Acq_Duration = 1.9970676[s]
X_Domain = Proton
X_Freq = 399.78219838[MHz]
X_Offset = 5[ppm]
X_Points = 14993
X_Points_Input = 11994
X_Prescans = 1
X_Resolution = 0.50073418[Hz]
X_Sweep = 7.50750751[kHz]
X_Sweep_Clipped = 6.00600601[kHz]
X_Sweep_Input = 15[ppm]
Irr_Domain = Proton
Irr_Freq = 399.78219838[MHz]
Irr_Offset = 5[ppm]
Tri_Domain = Proton
Tri_Freq = 399.78219838[MHz]
Tri_Offset = 5[ppm]
Blanking = 2[us]
Clipped = FALSE
Scans = 64
Total_Scans = 64
Relaxation_Delay = 5[s]
Recvr_Gain = 26
Temp_Get = 21.6[dC]
X_90_Width = 6.57[us]
X_Acq_Time = 1.9970676[s]
X_Angle = 45[deg]
X_Atn = 8[dB]
X_Data_Points = 65536
X_Points_Default = 25026
X_Pulse = 3.285[us]
Irr_Mode = Off
Tri_Mode = Off
Dante_Loop = 500
Dante_Presat = FALSE
JEOL
7.599 7.581 7.575 7.438 7.433 7.427 7.421 7.419 7.415 7.408 7.403 7.353 7.347 7.260 7.246 7.079 7.044 7.041 7.026 7.023
6.294 6.291 6.274 6.271
3.148 3.128 3.110 3.091 2.922 2.907 2.905 2.899 2.883 2.879 2.867 2.859 2.847 2.841 2.825 2.821 2.817 2.802
N
O
Cl
4b
abundance
0 0.1 0.2 0.3
9.0 8.0 7.0 6.0 5.0 4.0 3.0 2.0 1.0
1.00 2.00 1.00 1.00 2.00 1.00 2.00 2.00
X : parts per Million : Proton

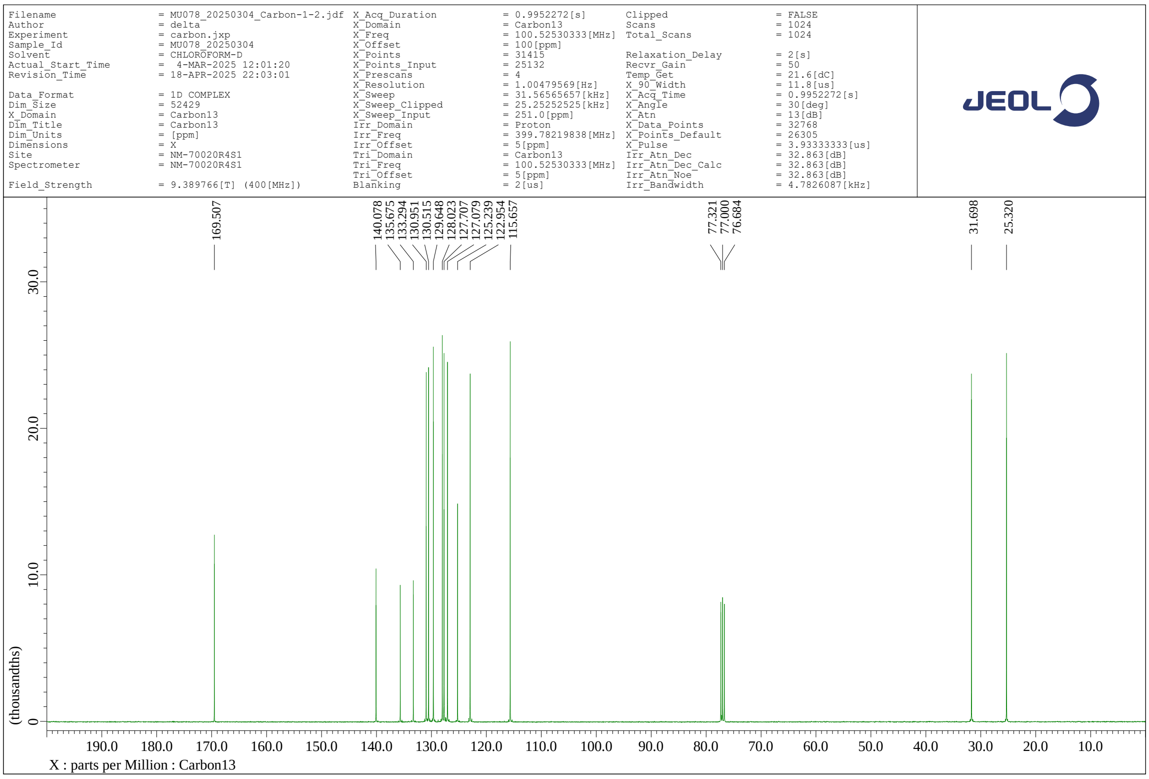
Filename = MU078_20250304_Carbon-1-2.jdf
Author = delta
Experiment = carbon.jxp
Sample_Id = MU078_20250304
Solvent = CHLOROFORM-D
Actual_Start_Time = 4-MAR-2025 12:01:20
Revision_Time = 18-APR-2025 22:03:01
Data_Format = 1D COMPLEX
Dim_Size = 52429
X_Domain = Carbon13
Dim_Title = Carbon13
Dim_Units = [ppm]
Dimensions = X
Site = NM-70020R4S1
Spectrometer = NM-70020R4S1
Field_Strength = 9.389766[T] (400[MHz])
X_Acq_Duration = 0.9952272[s]
X_Domain = Carbon13
X_Freq = 100.52530333[MHz]
X_Offset = 100[ppm]
X_Points = 31415
X_Points_Input = 25132
X_Prescans = 4
X_Resolution = 1.00479569[Hz]
X_Sweep = 31.56565657[kHz]
X_Sweep_Clipped = 25.25252525[kHz]
X_Sweep_Input = 251.0[ppm]
Irr_Domain = Proton
Irr_Freq = 399.78219838[MHz]
Irr_Offset = 5[ppm]
Tri_Domain = Carbon13
Tri_Freq = 100.52530333[MHz]
Tri_Offset = 5[ppm]
Blanking = 2[us]
Clipped = FALSE
Scans = 1024
Total_Scans = 1024
Relaxation_Delay = 2[s]
Recvr_Gain = 50
Temp_Get = 21.6[dC]
X_90_Width = 11.8[us]
X_Acq_Time = 0.9952272[s]
X_Angle = 30[deg]
X_Atn = 13[dB]
X_Data_Points = 32768
X_Points_Default = 26305
X_Pulse = 3.93333333[us]
Irr_Atn_Dec = 32.863[dB]
Irr_Atn_Dec_Calc = 32.863[dB]
Irr_Atn_Noe = 32.863[dB]
Irr_Bandwidth = 4.7826087[kHz]
JEOL
169.507
140.078 135.675 133.294 130.951 130.515 129.648 128.023 127.707 127.079 125.239 122.954 115.657
77.321 77.000 76.684
31.698
25.320
(thousandths)
0 10.0 20.0 30.0
190.0 180.0 170.0 160.0 150.0 140.0 130.0 120.0 110.0 100.0 90.0 80.0 70.0 60.0 50.0 40.0 30.0 20.0 10.0
X : parts per Million : Carbon13

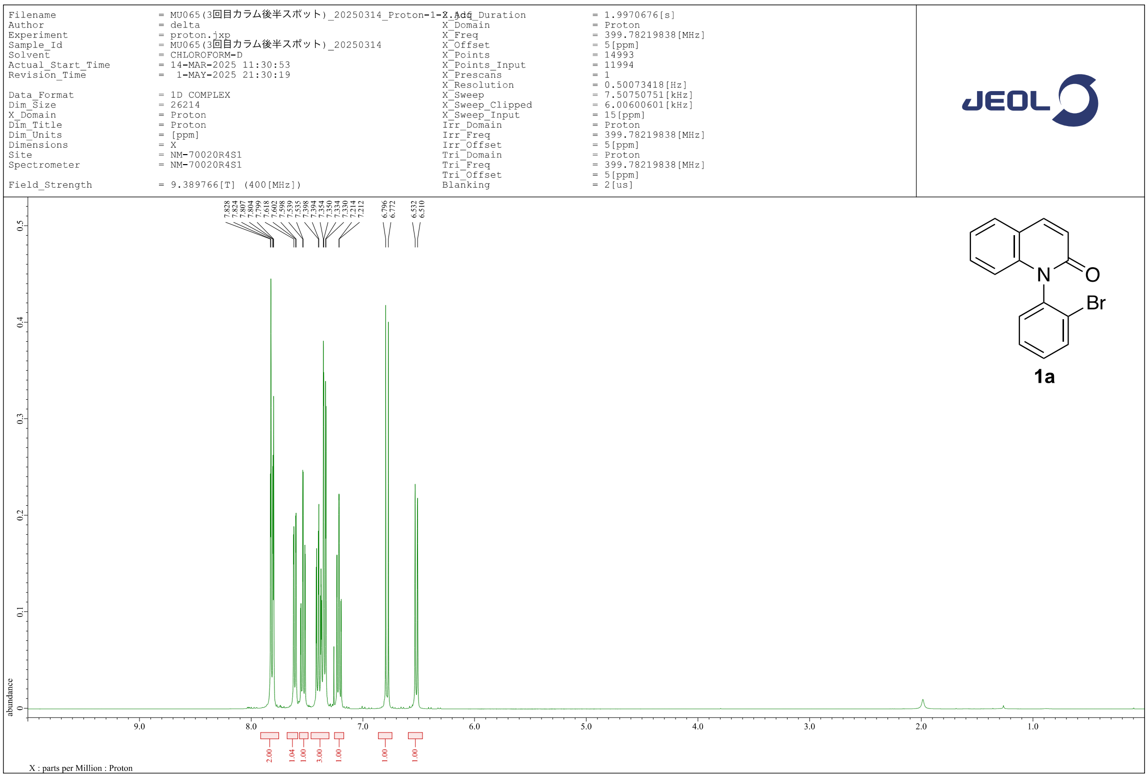
Filename = MU065(3回目カラム後半スポット)_20250314_Proton-1-
Author = delta
Experiment = proton.jxp
Sample_Id = MU065(3回目カラム後半スポット)_20250314
Solvent = CHLOROFORM-D
Actual_Start_Time = 14-MAR-2025 11:30:53
Revision_Time = 1-MAY-2025 21:30:19
Data_Format = 1D COMPLEX
Dim_Size = 26214
X_Domain = Proton
Dim_Title = Proton
Dim_Units = [ppm]
Dimensions = X
Site = NM-70020R4S1
Spectrometer = NM-70020R4S1
Field_Strength = 9.389766[T] (400[MHz])
X_Acq_Duration = 1.9970676[s]
X_Domain = Proton
X_Freq = 399.78219838[MHz]
X_Offset = 5[ppm]
X_Points = 14993
X_Points_Input = 11994
X_Prescans = 1
X_Resolution = 0.50073418[Hz]
X_Sweep = 7.50750751[kHz]
X_Sweep_Clipped = 6.00600601[kHz]
X_Sweep_Input = 15[ppm]
Irr_Domain = Proton
Irr_Freq = 399.78219838[MHz]
Irr_Offset = 5[ppm]
Tri_Domain = Proton
Tri_Freq = 399.78219838[MHz]
Tri_Offset = 5[ppm]
Blanking = 2[us]
JEOL
7.828 7.824 7.807 7.804 7.799 7.618 7.602 7.598 7.539 7.535 7.398 7.394 7.354 7.350 7.334 7.330 7.214 7.212 6.796 6.772 6.532 6.510
N
O
Br
1a
abundance
0 0.1 0.2 0.3 0.4 0.5
9.0 8.0 7.0 6.0 5.0 4.0 3.0 2.0 1.0
2.00 1.04 1.00 3.00 1.00 1.00 1.00
X : parts per Million : Proton

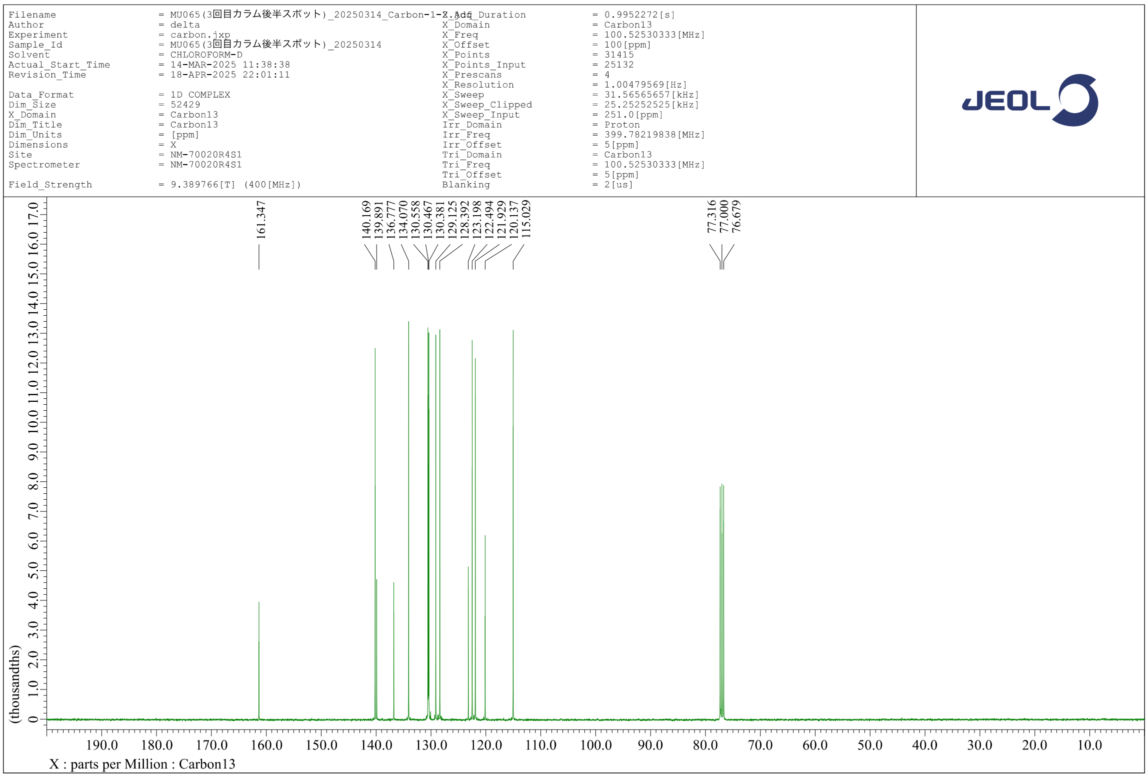
Filename = MU065(3回目カラム後半スポット)_20250314_Carbon-1-
Author = delta
Experiment = carbon.jxp
Sample_Id = MU065(3回目カラム後半スポット)_20250314
Solvent = CHLOROFORM-D
Actual_Start_Time = 14-MAR-2025 11:38:38
Revision_Time = 18-APR-2025 22:01:11
Data_Format = 1D COMPLEX
Dim_Size = 52429
X_Domain = Carbon13
Dim_Title = Carbon13
Dim_Units = [ppm]
Dimensions = X
Site = NM-70020R4S1
Spectrometer = NM-70020R4S1
Field_Strength = 9.389766[T] (400[MHz])
X_Acq_Duration = 0.9952272[s]
X_Domain = Carbon13
X_Freq = 100.52530333[MHz]
X_Offset = 100[ppm]
X_Points = 31415
X_Points_Input = 25132
X_Prescans = 4
X_Resolution = 1.00479569[Hz]
X_Sweep = 31.56565657[kHz]
X_Sweep_Clipped = 25.25252525[kHz]
X_Sweep_Input = 251.0[ppm]
Irr_Domain = Proton
Irr_Freq = 399.78219838[MHz]
Irr_Offset = 5[ppm]
Tri_Domain = Carbon13
Tri_Freq = 100.52530333[MHz]
Tri_Offset = 5[ppm]
Blanking = 2[us]
JEOL
161.347
140.169 139.891 136.777 134.070 130.558 130.467 130.381 129.125 128.392 123.198 122.494 121.929 120.137 115.029
77.316 77.000 76.679
(thousandths)
0 1.0 2.0 3.0 4.0 5.0 6.0 7.0 8.0 9.0 10.0 11.0 12.0 13.0 14.0 15.0 16.0 17.0
190.0 180.0 170.0 160.0 150.0 140.0 130.0 120.0 110.0 100.0 90.0 80.0 70.0 60.0 50.0 40.0 30.0 20.0 10.0
X : parts per Million : Carbon13

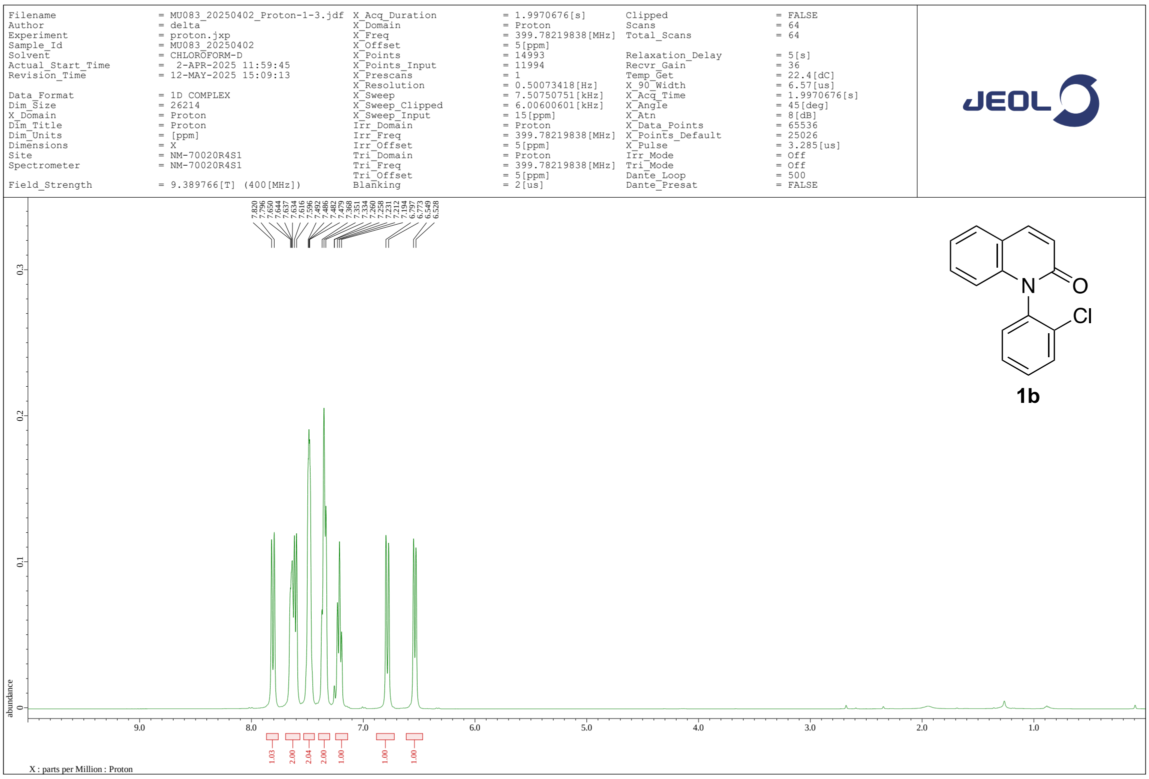
Filename = MU083_20250402_Proton-1-3.jdf
Author = delta
Experiment = proton.jxp
Sample_Id = MU083_20250402
Solvent = CHLOROFORM-D
Actual_Start_Time = 2-APR-2025 11:59:45
Revision_Time = 12-MAY-2025 15:09:13
Data_Format = 1D COMPLEX
Dim_Size = 26214
X_Domain = Proton
Dim_Title = Proton
Dim_Units = [ppm]
Dimensions = X
Site = NM-70020R4S1
Spectrometer = NM-70020R4S1
Field_Strength = 9.389766[T] (400[MHz])
X_Acq_Duration = 1.9970676[s]
X_Domain = Proton
X_Freq = 399.78219838[MHz]
X_Offset = 5[ppm]
X_Points = 14993
X_Points_Input = 11994
X_Prescans = 1
X_Resolution = 0.50073418[Hz]
X_Sweep = 7.50750751[kHz]
X_Sweep_Clipped = 6.00600601[kHz]
X_Sweep_Input = 15[ppm]
Irr_Domain = Proton
Irr_Freq = 399.78219838[MHz]
Irr_Offset = 5[ppm]
Tri_Domain = Proton
Tri_Freq = 399.78219838[MHz]
Tri_Offset = 5[ppm]
Blanking = 2[us]
Clipped = FALSE
Scans = 64
Total_Scans = 64
Relaxation_Delay = 5[s]
Recvr_Gain = 36
Temp_Get = 22.4[dC]
X_90_Width = 6.57[us]
X_Acq_Time = 1.9970676[s]
X_Angle = 45[deg]
X_Atn = 8[dB]
X_Data_Points = 65536
X_Points_Default = 25026
X_Pulse = 3.285[us]
Irr_Mode = Off
Tri_Mode = Off
Dante_Loop = 500
Dante_Presat = FALSE
JEOL
7.820 7.796 7.650 7.644 7.637 7.634 7.616 7.596 7.492 7.486 7.482 7.479 7.368 7.351 7.334 7.260 7.258 7.231 7.212 7.194 6.797 6.773 6.549 6.528
N
O
Cl
1b
abundance
0 0.1 0.2 0.3
9.0 8.0 7.0 6.0 5.0 4.0 3.0 2.0 1.0
1.03 2.00 2.04 2.00 1.00 1.00 1.00
X : parts per Million : Proton

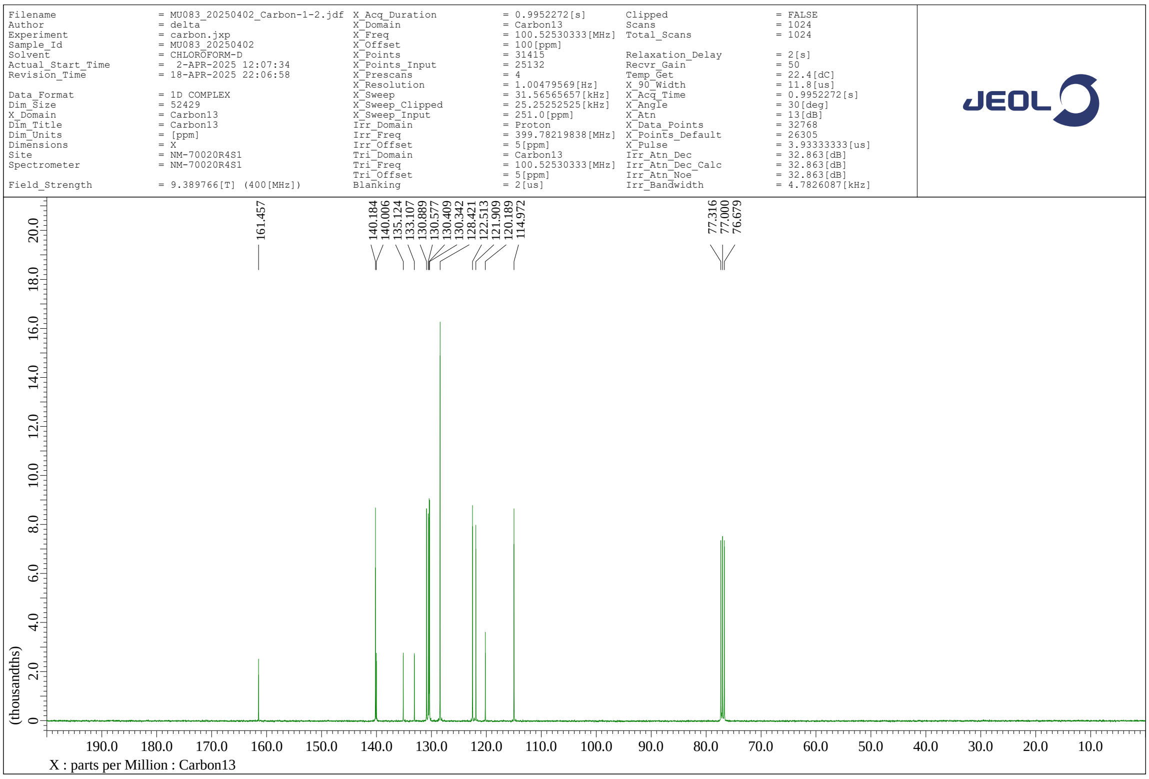
Filename = MU083_20250402_Carbon-1-2.jdf
Author = delta
Experiment = carbon.jxp
Sample_Id = MU083_20250402
Solvent = CHLOROFORM-D
Actual_Start_Time = 2-APR-2025 12:07:34
Revision_Time = 18-APR-2025 22:06:58
Data_Format = 1D COMPLEX
Dim_Size = 52429
X_Domain = Carbon13
Dim_Title = Carbon13
Dim_Units = [ppm]
Dimensions = X
Site = NM-70020R4S1
Spectrometer = NM-70020R4S1
Field_Strength = 9.389766[T] (400[MHz])
X_Acq_Duration = 0.9952272[s]
X_Domain = Carbon13
X_Freq = 100.52530333[MHz]
X_Offset = 100[ppm]
X_Points = 31415
X_Points_Input = 25132
X_Prescans = 4
X_Resolution = 1.00479569[Hz]
X_Sweep = 31.56565657[kHz]
X_Sweep_Clipped = 25.25252525[kHz]
X_Sweep_Input = 251.0[ppm]
Irr_Domain = Proton
Irr_Freq = 399.78219838[MHz]
Irr_Offset = 5[ppm]
Tri_Domain = Carbon13
Tri_Freq = 100.52530333[MHz]
Tri_Offset = 5[ppm]
Blanking = 2[us]
Clipped = FALSE
Scans = 1024
Total_Scans = 1024
Relaxation_Delay = 2[s]
Recvr_Gain = 50
Temp_Get = 22.4[dC]
X_90_Width = 11.8[us]
X_Acq_Time = 0.9952272[s]
X_Angle = 30[deg]
X_Atn = 13[dB]
X_Data_Points = 32768
X_Points_Default = 26305
X_Pulse = 3.93333333[us]
Irr_Atn_Dec = 32.863[dB]
Irr_Atn_Dec_Calc = 32.863[dB]
Irr_Atn_Noe = 32.863[dB]
Irr_Bandwidth = 4.7826087[kHz]
JEOL
161.457
140.184 140.006 135.124 133.107 130.889 130.577 130.409 130.342 128.421 122.513 121.909 120.189 114.972
77.316 77.000 76.679
(thousandths)
0 2.0 4.0 6.0 8.0 10.0 12.0 14.0 16.0 18.0 20.0
190.0 180.0 170.0 160.0 150.0 140.0 130.0 120.0 110.0 100.0 90.0 80.0 70.0 60.0 50.0 40.0 30.0 20.0 10.0
X : parts per Million : Carbon13

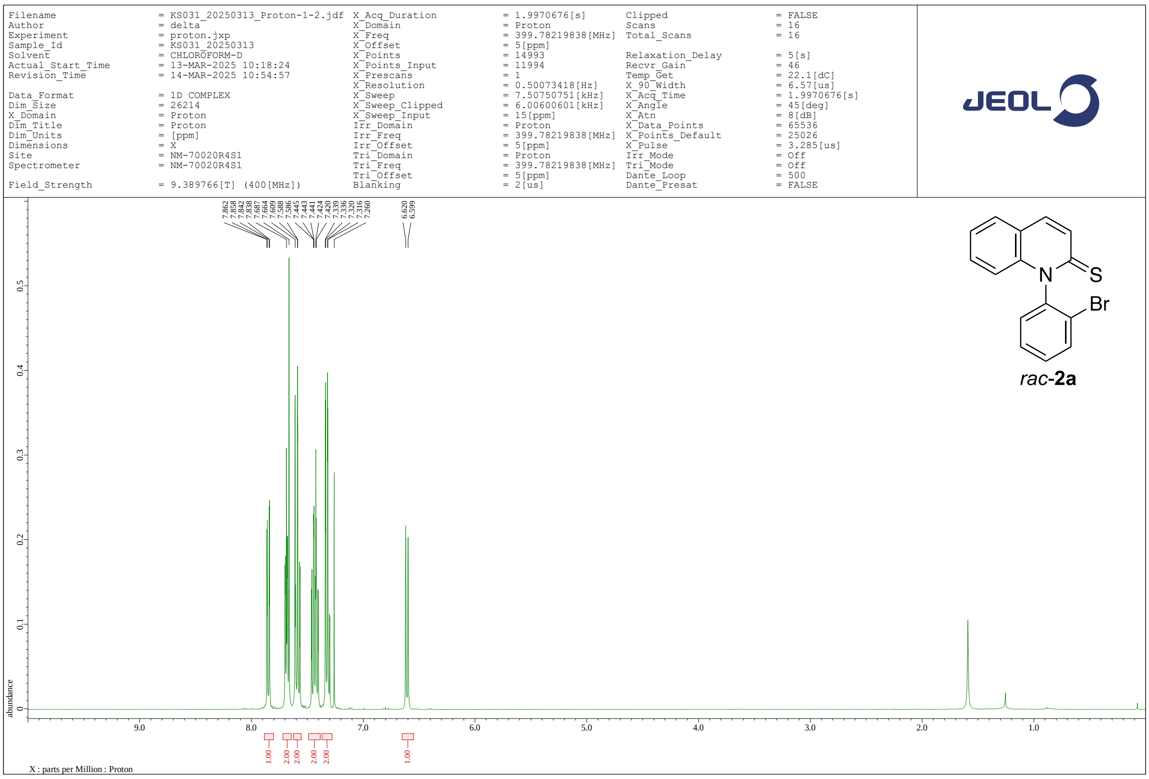
Filename = KS031_20250313_Proton-1-2.jdf
Author = delta
Experiment = proton.jxp
Sample_Id = KS031_20250313
Solvent = CHLOROFORM-D
Actual_Start_Time = 13-MAR-2025 10:18:24
Revision_Time = 14-MAR-2025 10:54:57
Data_Format = 1D COMPLEX
Dim_Size = 26214
X_Domain = Proton
Dim_Title = Proton
Dim_Units = [ppm]
Dimensions = X
Site = NM-70020R4S1
Spectrometer = NM-70020R4S1
Field_Strength = 9.389766[T] (400[MHz])
X_Acq_Duration = 1.9970676[s]
X_Domain = Proton
X_Freq = 399.78219838[MHz]
X_Offset = 5[ppm]
X_Points = 14993
X_Points_Input = 11994
X_Prescans = 1
X_Resolution = 0.50073418[Hz]
X_Sweep = 7.50750751[kHz]
X_Sweep_Clipped = 6.00600601[kHz]
X_Sweep_Input = 15[ppm]
Irr_Domain = Proton
Irr_Freq = 399.78219838[MHz]
Irr_Offset = 5[ppm]
Tri_Domain = Proton
Tri_Freq = 399.78219838[MHz]
Tri_Offset = 5[ppm]
Blanking = 2[us]
Clipped = FALSE
Scans = 16
Total_Scans = 16
Relaxation_Delay = 5[s]
Recvr_Gain = 46
Temp_Get = 22.1[dC]
X_90_Width = 6.57[us]
X_Acq_Time = 1.9970676[s]
X_Angle = 45[deg]
X_Atn = 8[dB]
X_Data_Points = 65536
X_Points_Default = 25026
X_Pulse = 3.285[us]
Irr_Mode = Off
Tri_Mode = Off
Dante_Loop = 500
Dante_Presat = FALSE
JEOL
S
N
Br
rac-2a
7.862 7.858 7.842 7.838 7.687 7.664 7.609 7.590 7.586 7.445 7.443 7.441 7.424 7.420 7.339 7.336 7.320 7.316 7.260
6.620 6.599
1.00 2.00 2.00 2.00 2.00 1.00
abundance
X : parts per Million : Proton

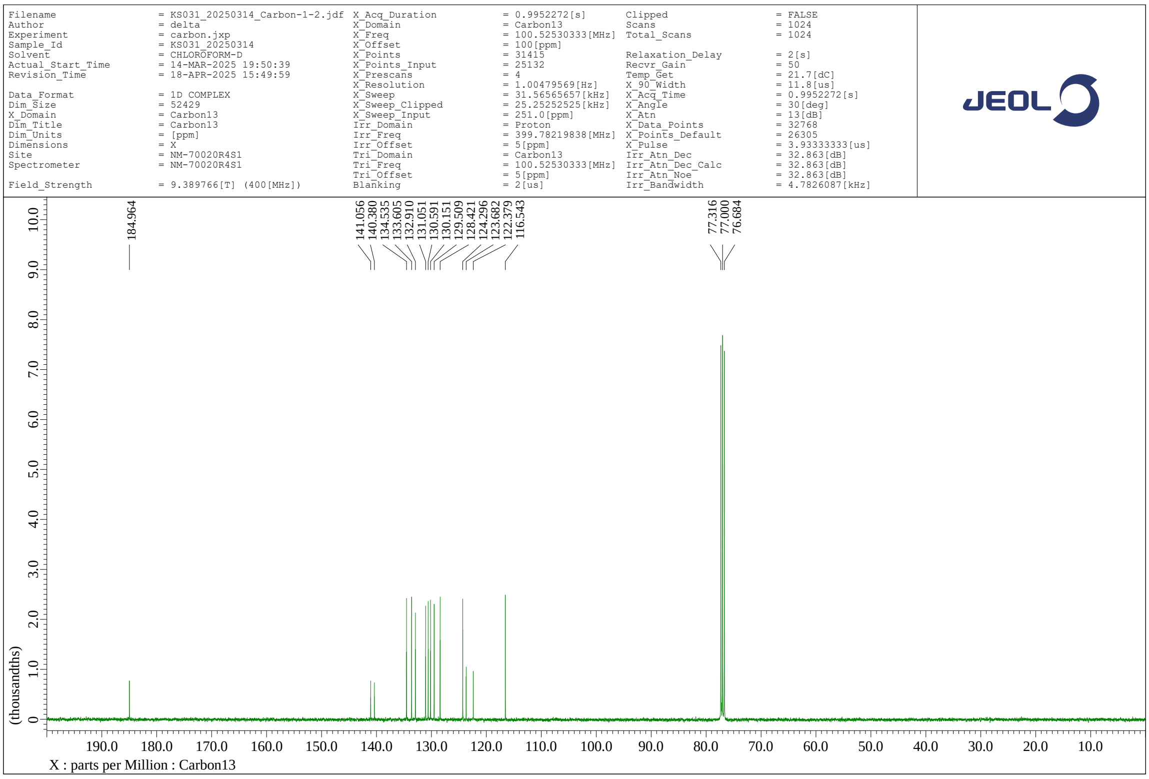
Filename = KS031_20250314_Carbon-1-2.jdf
Author = delta
Experiment = carbon.jxp
Sample_Id = KS031_20250314
Solvent = CHLOROFORM-D
Actual_Start_Time = 14-MAR-2025 19:50:39
Revision_Time = 18-APR-2025 15:49:59
Data_Format = 1D COMPLEX
Dim_Size = 52429
X_Domain = Carbon13
Dim_Title = Carbon13
Dim_Units = [ppm]
Dimensions = X
Site = NM-70020R4S1
Spectrometer = NM-70020R4S1
Field_Strength = 9.389766[T] (400[MHz])
X_Acq_Duration = 0.9952272[s]
X_Domain = Carbon13
X_Freq = 100.52530333[MHz]
X_Offset = 100[ppm]
X_Points = 31415
X_Points_Input = 25132
X_Prescans = 4
X_Resolution = 1.00479569[Hz]
X_Sweep = 31.56565657[kHz]
X_Sweep_Clipped = 25.25252525[kHz]
X_Sweep_Input = 251.0[ppm]
Irr_Domain = Proton
Irr_Freq = 399.78219838[MHz]
Irr_Offset = 5[ppm]
Tri_Domain = Carbon13
Tri_Freq = 100.52530333[MHz]
Tri_Offset = 5[ppm]
Blanking = 2[us]
Clipped = FALSE
Scans = 1024
Total_Scans = 1024
Relaxation_Delay = 2[s]
Recvr_Gain = 50
Temp_Get = 21.7[dC]
X_90_Width = 11.8[us]
X_Acq_Time = 0.9952272[s]
X_Angle = 30[deg]
X_Atn = 13[dB]
X_Data_Points = 32768
X_Points_Default = 26305
X_Pulse = 3.93333333[us]
Irr_Atn_Dec = 32.863[dB]
Irr_Atn_Dec_Calc = 32.863[dB]
Irr_Atn_Noe = 32.863[dB]
Irr_Bandwidth = 4.7826087[kHz]
JEOL
184.964
141.056 140.380 134.535 133.605 132.910 131.051 130.591 130.151 129.509 128.421 124.296 123.682 122.379 116.543
77.316 77.000 76.684
(thousandths)
X : parts per Million : Carbon13

Chiral MPLC and HPLC charts of compounds 1a,b and 2a

Enantiomer separation through MPLC using a semi-preparative chiral IH column

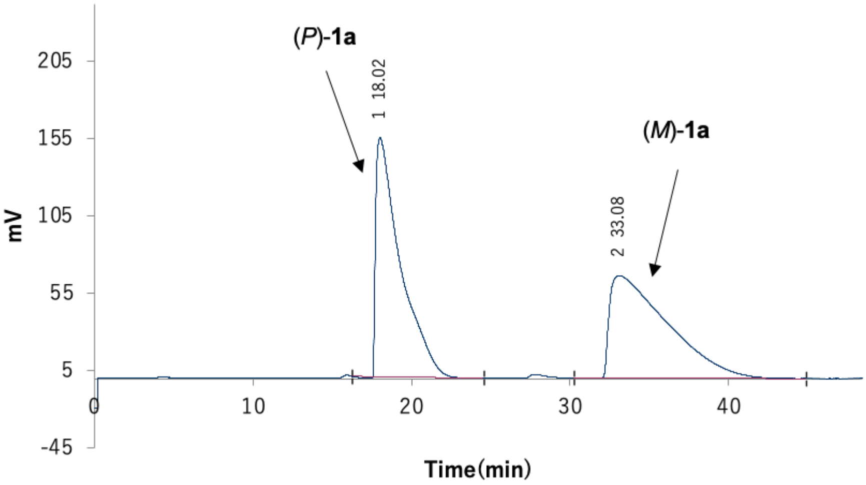

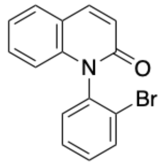

rac-**1a**

IH
eluent: 15% IPA in hexane
flow rate: 20×0.2 mL/min

No.	Rt	Area	Area(%)	Height
1	18.02	16557699	49.5335	154433
2	33.08	16869591	50.4665	66430
		33427290	100	220863

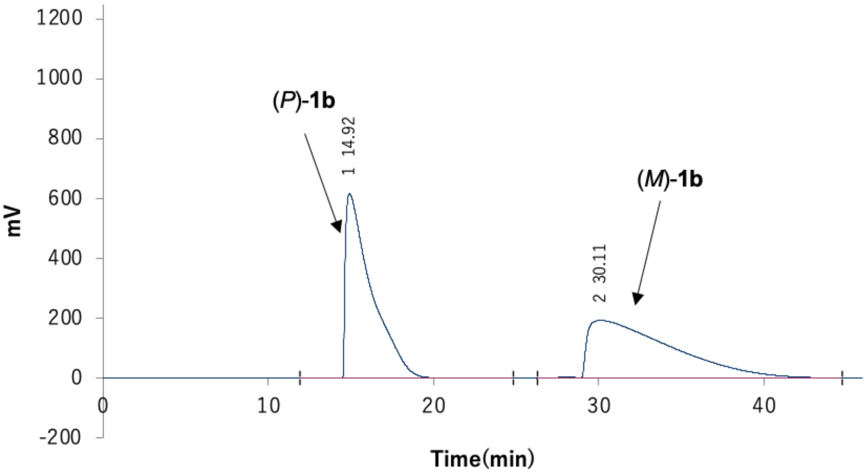

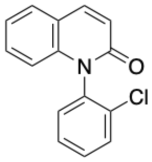

rac-**1b**

IH
eluent: 15% IPA in hexane
flow rate: 20×0.2 mL/min

No.	Rt	Area	Area(%)	Height
1	14.92	67191427	49.7992	615786
2	30.11	67733376	50.2008	192323
		1.35E+08	100	808109

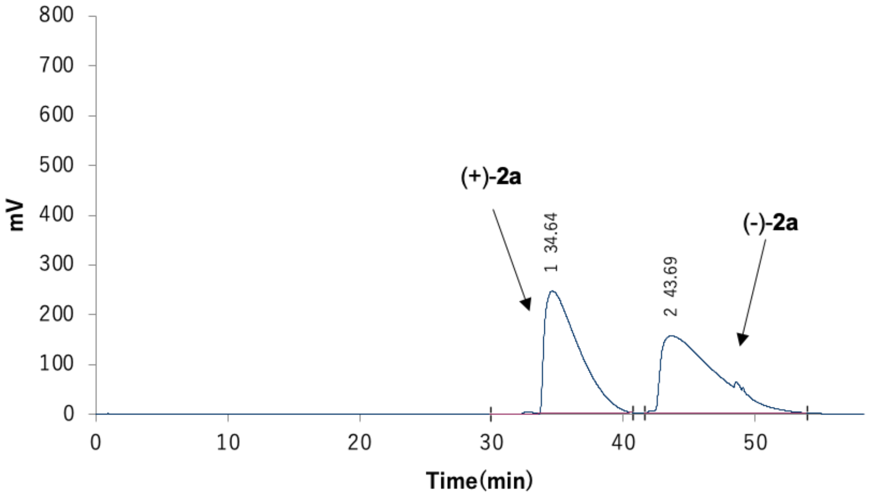

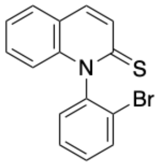

rac-**2a**

IH
eluent: 5% IPA in hexane
flow rate: 20×0.2 mL/min

No.	Rt	Area	Area(%)	Height
1	34.64	44231636	49.7262	245539
2	43.69	44718717	50.2738	155483
		88950353	100	401022

HPLC analysis using a chiral column for the determination of ee

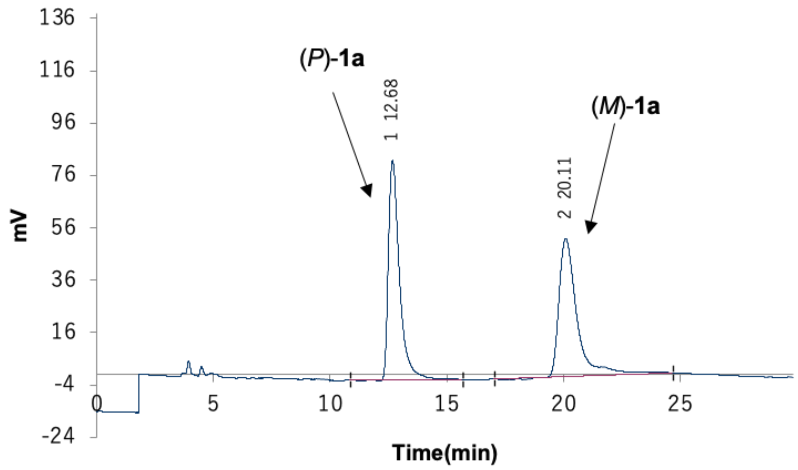

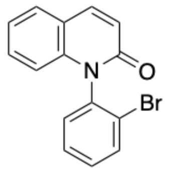

AD-H
eluent: 15% IPA in hexane
flow rate: 0.8 mL/min

No.	Rt	Area	Area(%)	Height
1	12.68	2769672	49.6187	83757
2	20.11	2812238	50.3813	52657
		5581910	100	136414

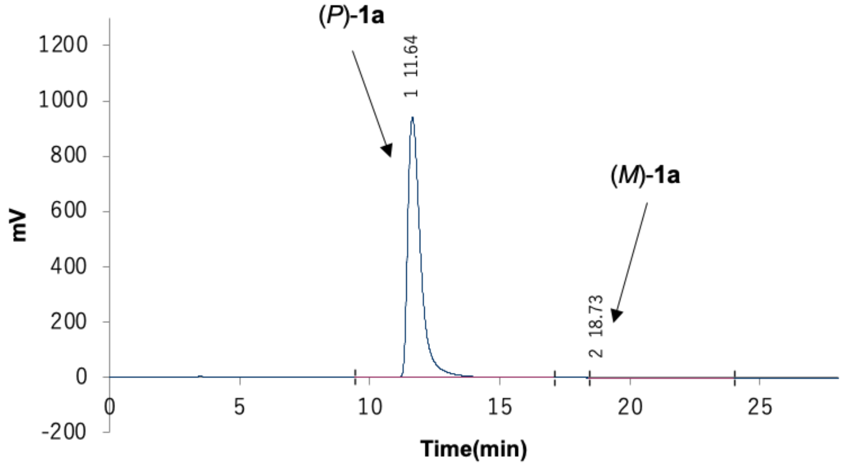

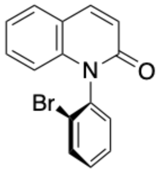

AD-H
eluent: 15% IPA in hexane
flow rate: 0.8 mL/min

No.	Rt	Area	Area(%)	Height
1	11.64	32319038	99.8511	941725
2	18.73	48207.2	0.1489	86
		32367245	100	941811

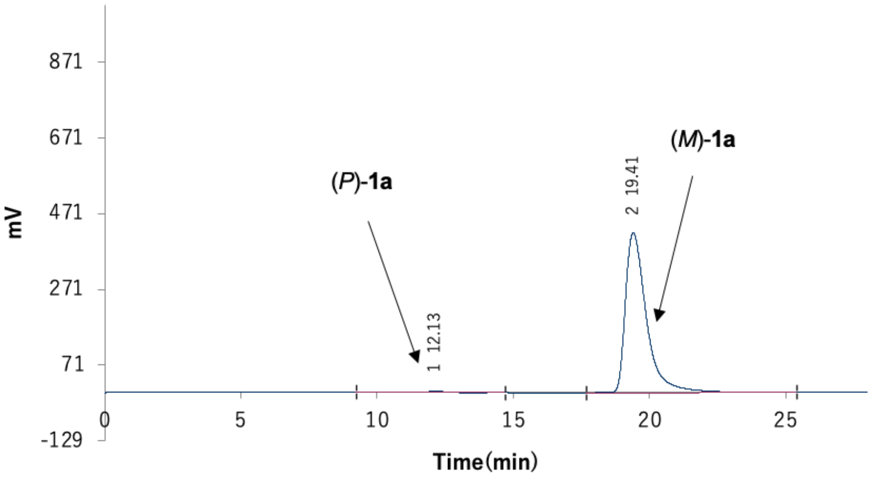

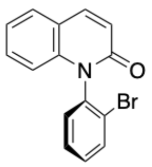

(*M*)-**1a**

AD-H
eluent: 15% IPA in hexane
flow rate: 0.8 mL/min

No.	Rt	Area	Area(%)	Height
1	12.13	78018.2	0.341	4961
2	19.41	22803947	99.659	422652
		22881965	100	427613

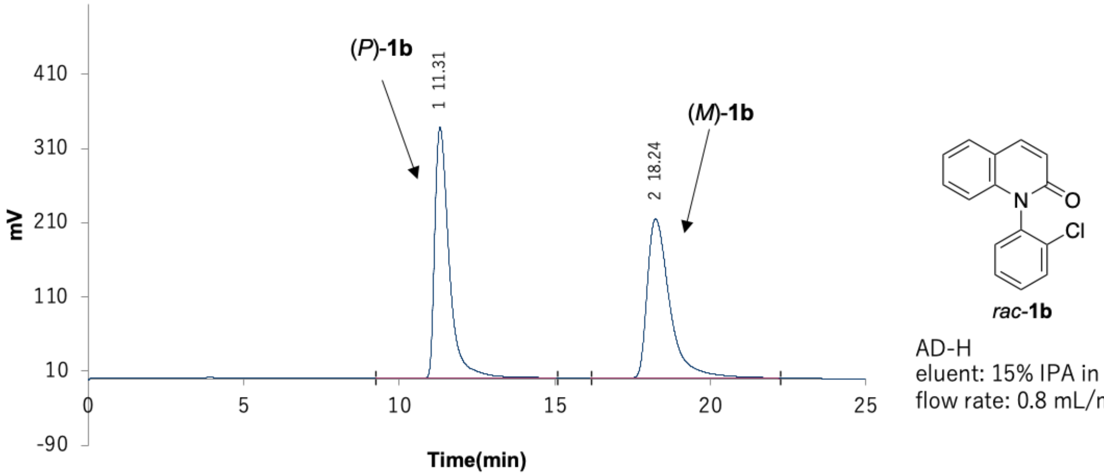

AD-H
eluent: 15% IPA in hexane
flow rate: 0.8 mL/min

No.	Rt	Area	Area(%)	Height
1	11.31	10815213	50.0329	337776
2	18.24	10800974	49.9671	214668
		21616186	100	552444

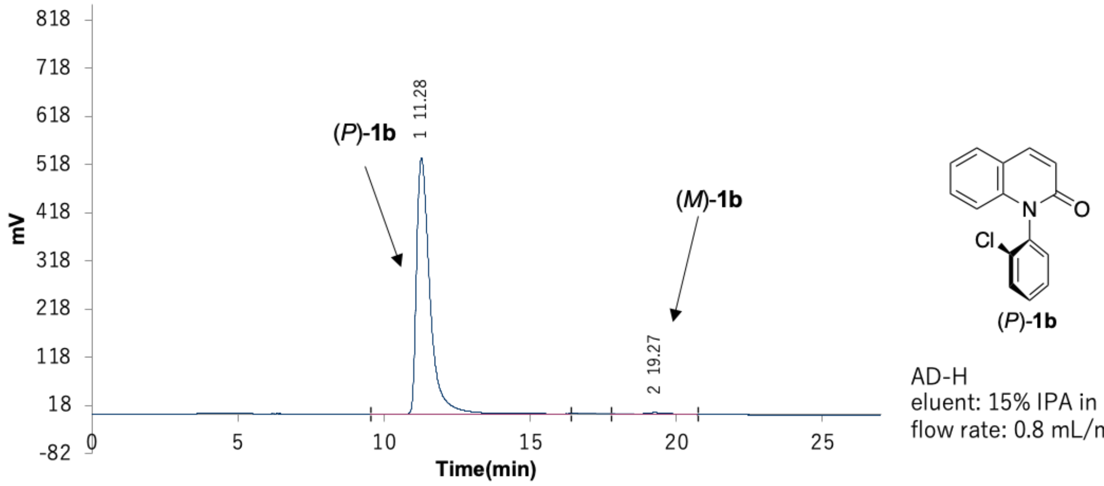

AD-H
eluent: 15% IPA in hexane
flow rate: 0.8 mL/min

No.	Rt	Area	Area(%)	Height
1	11.28	16876141	99.045	531326
2	19.27	162719.4	0.955	3151
		17038860	100	534477

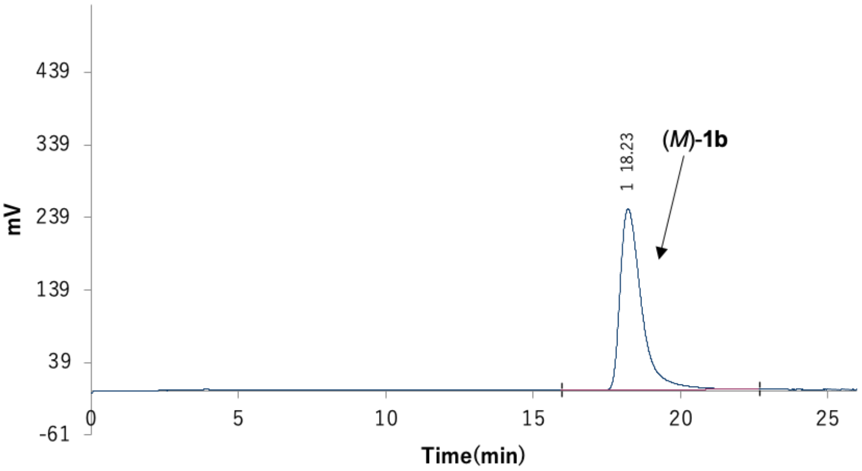

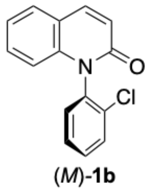

AD-H
eluent: 15% IPA in hexane
flow rate: 0.8 mL/min

No.	Rt	Area	Area(%)	Height
1	18.23	12517220	100	249364
		12517220	100	249364

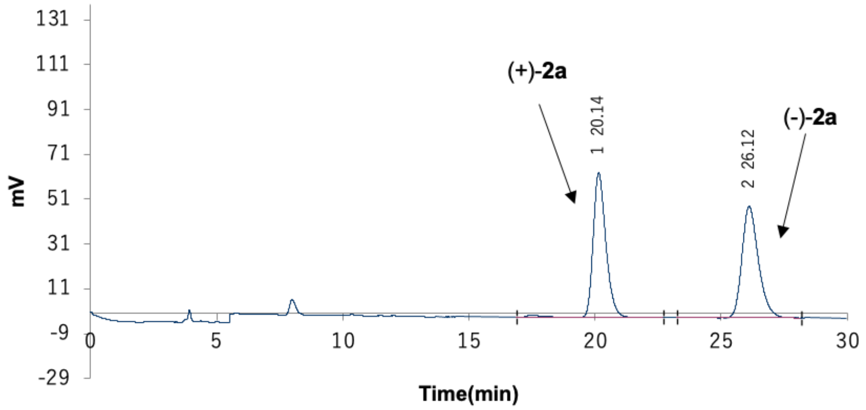

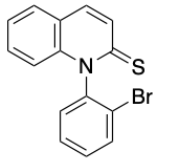

rac-**2a**

AS-H
eluent: 15% IPA in hexane
flow rate: 0.8 mL/min

No.	Rt	Area	Area(%)	Height
1	20.14	2279662.6	50.594	64540
2	26.12	2226131.5	49.406	49610
		4505794.1	100	114150

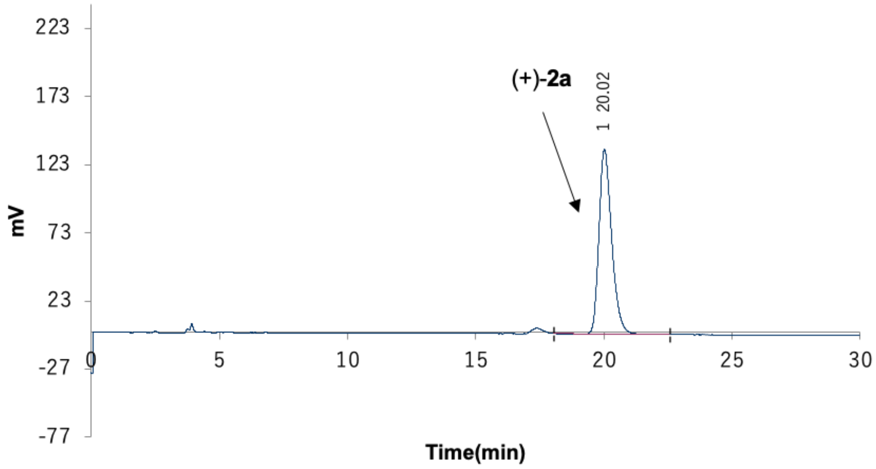

(+)-**2a**

AS-H
eluent: 15% IPA in hexane
flow rate: 0.8 mL/min

No.	Rt	Area	Area(%)	Height
1	20.02	4655608	100	135501
		4655608	100	135501

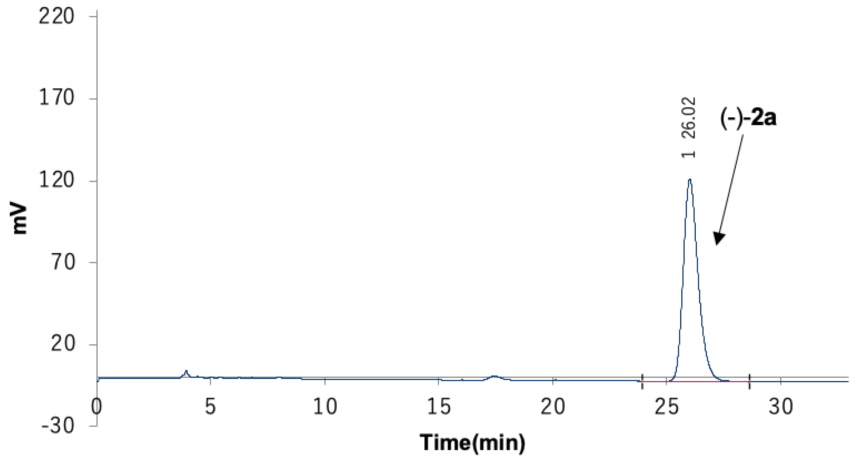

(-)-2a

AS-H
eluent: 15% IPA in hexane
flow rate: 0.8 mL/min

No.	Rt	Area	Area(%)	Height
1	26.02	5603254	100	123532
		5603254	100	123532

Evaluation of rotational barriers of compounds 1a and 1b

(Evaluation of rotational barrier of **1a** at 116 °C in 1,2-dichloroethane)

Time (s)	less polar(%)	more polar(%)	% ee
0	98.79	1.21	97.6
3600	95.37	4.63	90.7
7200	92.99	7.01	86.0
18000	85.35	14.65	70.7
37800	77.55	22.45	55.1
59400	66.61	33.39	33.2
72000	63.02	36.98	26.0
86400	60.39	39.61	20.8
111600	56.35	43.64	12.7
140400	53.91	46.09	7.8
154800	53.39	46.61	6.8
183600	53.37	46.63	6.7

Temp (°C)	T (K)	k (s^{-1})	$\Delta G^{\ddagger}$ (kcal/mol)
116	389.15	8	32.0

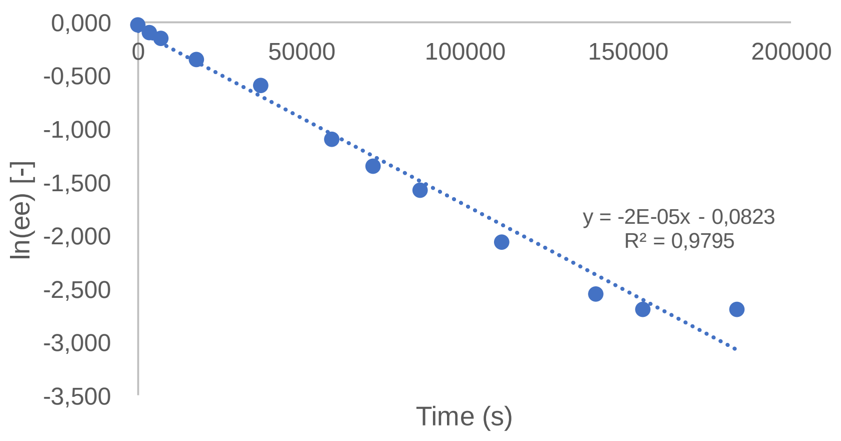

(Evaluation of rotational barrier of **1b** at 96 °C in 1,2-dichloroethane)

Time [s]	less polar(%)	more polar(%)	% ee
0	100	0	100
1800	99.06	0.94	98.1
3600	98.54	1.46	97.1
5400	97.75	2.25	95.5
7200	97.16	2.84	94.3
10800	95.73	4.27	91.5
21600	91.85	8.15	83.7
39600	83.81	16.19	67.6
54000	81.48	18.52	63
82800	73.66	26.34	47.3
147600	63.73	36.27	27.4

**Temp (°C)**	**T (K)**	**k (s^{-1})**	**$\Delta G^{\ddagger}$ (kcal/mol)**
96	369.15	8	30.8

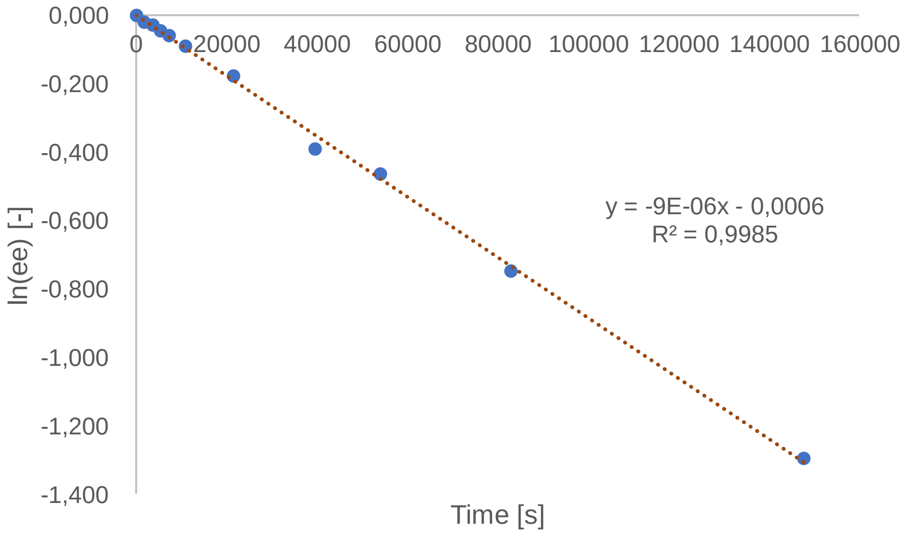

X-ray crystal data of *rac*-1a,b, (*P*)-1a,b, *rac*-2a (check CIF)

checkCIF/PLATON report

rac-1a

Structure factors have been supplied for datablock(s) TS221024A2-4_a

THIS REPORT IS FOR GUIDANCE ONLY. IF USED AS PART OF A REVIEW PROCEDURE FOR PUBLICATION, IT SHOULD NOT REPLACE THE EXPERTISE OF AN EXPERIENCED CRYSTALLOGRAPHIC REFEREE.

No syntax errors found. CIF dictionary Interpreting this report

Datablock: TS221024A2-4_a

Bond precision:	C-C = 0.0101 A	Wavelength=0.71073	
Cell:	a=16.488(2)	b=39.310(6)	c=7.5814(12)
	alpha=90	beta=90	gamma=90
Temperature:	100 K		

	Calculated	Reported
Volume	4913.8(12)	4913.8(13)
Space group	F d d 2	F d d 2
Hall group	F 2 -2d	F 2 -2d
Moiety formula	C15 H10 Br N O	C15 H10 Br N O
Sum formula	C15 H10 Br N O	C15 H10 Br N O
Mr	300.14	300.15
Dx,g cm-3	1.623	1.623
Z	16	16
Mu (mm-1)	3.331	3.331
F000	2400.0	2400.0
F000’	2396.35	
h,k,lmax	19,46,9	19,46,9
Nref	2165[1172]	2157
Tmin,Tmax	0.586,0.808	0.690,0.810
Tmin’	0.468	

Correction method= # Reported T Limits: Tmin=0.690 Tmax=0.810
AbsCorr = MULTI-SCAN

Data completeness= 1.84/1.00 Theta(max)= 25.018

R(reflections)= 0.0367(2032) wR2(reflections)= 0.0863(2157)

S = 1.084 Npar= 163

The following ALERTS were generated. Each ALERT has the format
test-name_ALERT_alert-type_alert-level.
Click on the hyperlinks for more details of the test.

Alert level C

PLAT090_ALERT_3_C Poor Data / Parameter Ratio (Zmax > 18) .... 7.18 Note
PLAT213_ALERT_2_C Atom C15 has ADP max/min Ratio ..... 3.9 prolat
PLAT341_ALERT_3_C Low Bond Precision on C-C Bonds .... 0.01007 Ang.
PLAT927_ALERT_1_C Reported and Calculated wR2 Differ by .... 0.0012 Check
PLAT987_ALERT_1_C The Flack x is >> 0 - Do a BASF/TWIN Refinement Please Check

Alert level G

PLAT083_ALERT_2_G SHELXL Second Parameter in WGHT Unusually Large 35.95 Why ?
PLAT432_ALERT_2_G Short Inter X...Y Contact Br1 ..C6 . 3.30 Ang.
x,y,-1+z = 1_554 Check
PLAT883_ALERT_1_G Absent Datum for _atom_sites_solution_primary .. Please Do !
PLAT899_ALERT_4_G SHELXL2018 is Outdated and Succeeded by SHELXL 2019/3 Note
PLAT909_ALERT_3_G Percentage of I>2sig(I) Data at Theta(Max) Still 85% Note
PLAT910_ALERT_3_G Missing # of FCF Reflection(s) Below Theta(Min). 1 Note
0 4 0,
PLAT965_ALERT_2_G The SHELXL WEIGHT Optimisation has not Converged Please Check
PLAT969_ALERT_5_G The 'Henn et al.' R-Factor-gap value .... 1.394 Note
Predicted wR2: Based on SigI**2 6.19 or SHELX Weight 7.96
PLAT978_ALERT_2_G Number C-C Bonds with Positive Residual Density. 1 Info

0 **ALERT level A** = Most likely a serious problem - resolve or explain
0 **ALERT level B** = A potentially serious problem, consider carefully
5 **ALERT level C** = Check. Ensure it is not caused by an omission or oversight
9 **ALERT level G** = General information/check it is not something unexpected

3 ALERT type 1 CIF construction/syntax error, inconsistent or missing data
5 ALERT type 2 Indicator that the structure model may be wrong or deficient
4 ALERT type 3 Indicator that the structure quality may be low
1 ALERT type 4 Improvement, methodology, query or suggestion
1 ALERT type 5 Informative message, check

It is advisable to attempt to resolve as many as possible of the alerts in all categories. Often the minor alerts point to easily fixed oversights, errors and omissions in your CIF or refinement strategy, so attention to these fine details can be worthwhile. In order to resolve some of the more serious problems it may be necessary to carry out additional measurements or structure refinements. However, the purpose of your study may justify the reported deviations and the more serious of these should normally be commented upon in the discussion or experimental section of a paper or in the "special_details" fields of the CIF. checkCIF was carefully designed to identify outliers and unusual parameters, but every test has its limitations and alerts that are not important in a particular case may appear. Conversely, the absence of alerts does not guarantee there are no aspects of the results needing attention. It is up to the individual to critically assess their own results and, if necessary, seek expert advice.

Publication of your CIF in IUCr journals

A basic structural check has been run on your CIF. These basic checks will be run on all CIFs submitted for publication in IUCr journals (*Acta Crystallographica*, *Journal of Applied Crystallography*, *Journal of Synchrotron Radiation*); however, if you intend to submit to *Acta Crystallographica Section C* or *E* or *IUCrData*, you should make sure that full publication checks are run on the final version of your CIF prior to submission.

Publication of your CIF in other journals

Please refer to the *Notes for Authors* of the relevant journal for any special instructions relating to CIF submission.

PLATON version of 02/02/2025; check.def file version of 02/02/2025

Datablock TS221024A2-4_a - ellipsoid plot

ORTEP drawing of ***rac*-1a** showing thermal ellipsoids at the 50% probability level.

Single colorless crystals of ***rac*-1a** were obtained by vapour diffusion of hexane into a methanol solution of the compound at room temperature.

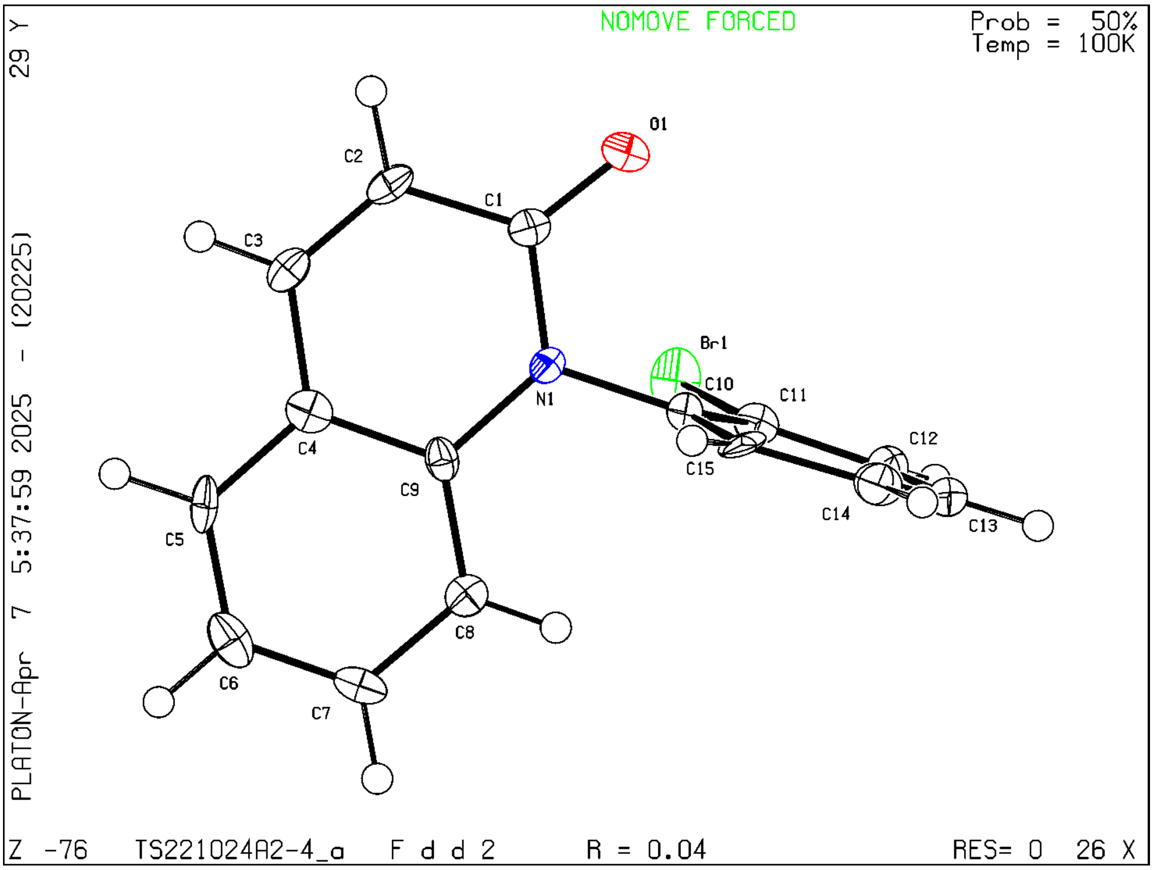

checkCIF/PLATON report

(*P*)-1a

Structure factors have been supplied for datablock(s) TS2211025B_0m_a

THIS REPORT IS FOR GUIDANCE ONLY. IF USED AS PART OF A REVIEW PROCEDURE FOR PUBLICATION, IT SHOULD NOT REPLACE THE EXPERTISE OF AN EXPERIENCED CRYSTALLOGRAPHIC REFEREE.

No syntax errors found. CIF dictionary Interpreting this report

Datablock: TS2211025B_0m_a

Bond precision:	C-C = 0.0033 A		Wavelength=0.71073
Cell:	a=7.2074(2)	b=7.7877(3)	c=22.6279(6)
	alpha=90	beta=90	gamma=90
Temperature:	100 K		

	Calculated	Reported
Volume	1270.08(7)	1270.08(7)
Space group	P 21 21 21	P 21 21 21
Hall group	P 2ac 2ab	P 2ac 2ab
Moiety formula	C15 H10 Br N O	C15 H10 Br N O
Sum formula	C15 H10 Br N O	C15 H10 Br N O
Mr	300.14	300.15
Dx,g cm-3	1.570	1.570
Z	4	4
Mu (mm-1)	3.222	3.222
F000	600.0	600.0
F000’	599.09	
h,k,lmax	8,9,26	8,9,26
Nref	2261[1340]	2256
Tmin,Tmax	0.569,0.660	0.620,0.680
Tmin’	0.558	

Correction method= # Reported T Limits: Tmin=0.620 Tmax=0.680
AbsCorr = MULTI-SCAN

Data completeness= 1.68/1.00 Theta(max)= 25.002

R(reflections)= 0.0166(2210) wR2(reflections)= 0.0376(2256)

S = 1.052 Npar= 163

The following ALERTS were generated. Each ALERT has the format
test-name_ALERT_alert-type_alert-level.
Click on the hyperlinks for more details of the test.

Alert level G

PLAT883_ALERT_1_G Absent Datum for _atom_sites_solution_primary ..	Please	Do !
PLAT899_ALERT_4_G SHELXL2018 is Outdated and Succeeded by SHELXL	2019/3	Note
PLAT909_ALERT_3_G Percentage of I>2sig(I) Data at Theta(Max) Still	94%	Note
PLAT910_ALERT_3_G Missing # of FCF Reflection(s) Below Theta(Min).	1	Note
0 0 2,		
PLAT969_ALERT_5_G The 'Henn et al.' R-Factor-gap value	1.117	Note
Predicted wR2: Based on SigI**2 3.36 or SHELX Weight 3.57		
PLAT978_ALERT_2_G Number C-C Bonds with Positive Residual Density.	9	Info

0 **ALERT level A** = Most likely a serious problem - resolve or explain
0 **ALERT level B** = A potentially serious problem, consider carefully
0 **ALERT level C** = Check. Ensure it is not caused by an omission or oversight
6 **ALERT level G** = General information/check it is not something unexpected

1 ALERT type 1 CIF construction/syntax error, inconsistent or missing data
1 ALERT type 2 Indicator that the structure model may be wrong or deficient
2 ALERT type 3 Indicator that the structure quality may be low
1 ALERT type 4 Improvement, methodology, query or suggestion
1 ALERT type 5 Informative message, check

It is advisable to attempt to resolve as many as possible of the alerts in all categories. Often the minor alerts point to easily fixed oversights, errors and omissions in your CIF or refinement strategy, so attention to these fine details can be worthwhile. In order to resolve some of the more serious problems it may be necessary to carry out additional measurements or structure refinements. However, the purpose of your study may justify the reported deviations and the more serious of these should normally be commented upon in the discussion or experimental section of a paper or in the "special_details" fields of the CIF. checkCIF was carefully designed to identify outliers and unusual parameters, but every test has its limitations and alerts that are not important in a particular case may appear. Conversely, the absence of alerts does not guarantee there are no aspects of the results needing attention. It is up to the individual to critically assess their own results and, if necessary, seek expert advice.

Publication of your CIF in IUCr journals

A basic structural check has been run on your CIF. These basic checks will be run on all CIFs submitted for publication in IUCr journals (*Acta Crystallographica*, *Journal of Applied Crystallography*, *Journal of Synchrotron Radiation*); however, if you intend to submit to *Acta Crystallographica Section C* or *E* or *IUCrData*, you should make sure that full publication checks are run on the final version of your CIF prior to submission.

Publication of your CIF in other journals

Please refer to the *Notes for Authors* of the relevant journal for any special instructions relating to CIF submission.

PLATON version of 02/02/2025; check.def file version of 02/02/2025

Datablock TS2211025B_0m_a - ellipsoid plot

ORTEP drawing of ***(P)*-1a** showing thermal ellipsoids at the 50% probability level.

Single colorless crystals of ***(P)*-1a** were obtained by vapour diffusion of hexane into a methanol solution of the compound at room temperature.

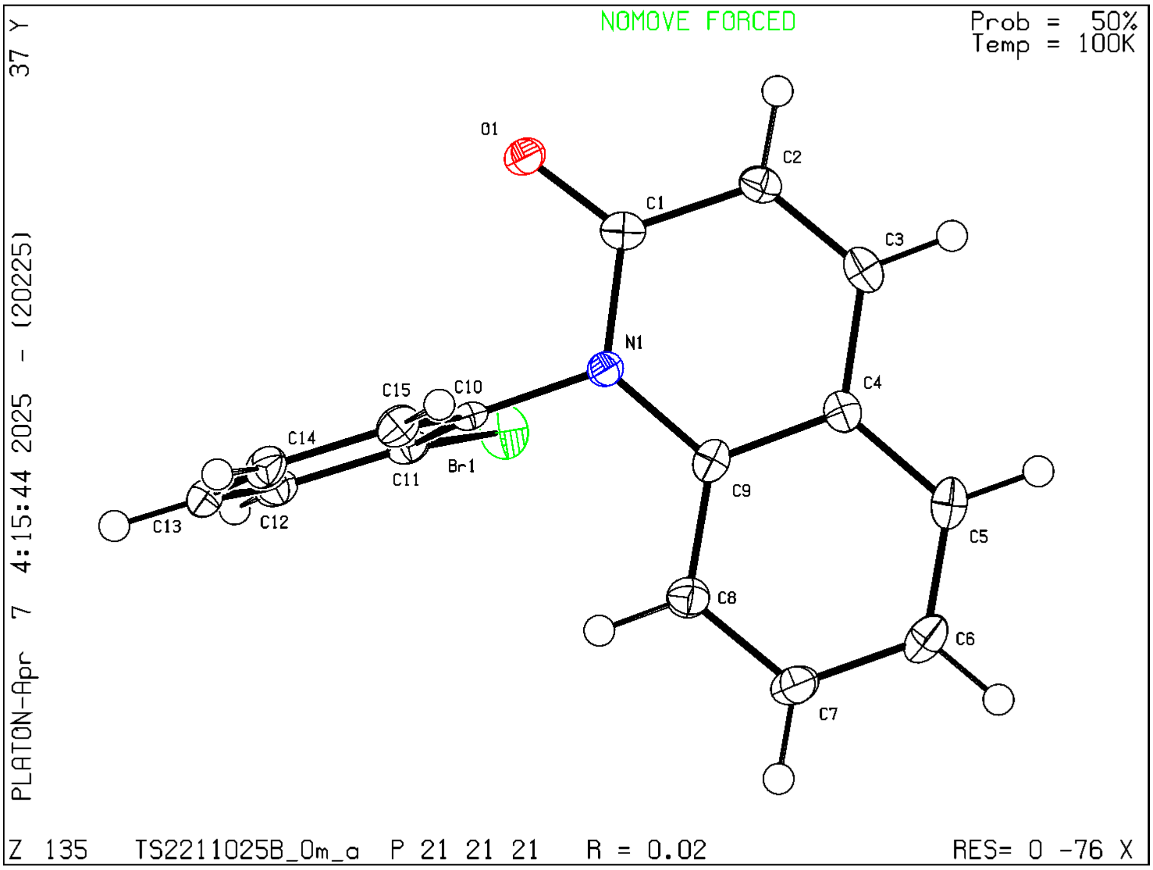

checkCIF/PLATON report

rac-1b

You have not supplied any structure factors. As a result the full set of tests cannot be run.

THIS REPORT IS FOR GUIDANCE ONLY. IF USED AS PART OF A REVIEW PROCEDURE FOR PUBLICATION, IT SHOULD NOT REPLACE THE EXPERTISE OF AN EXPERIENCED CRYSTALLOGRAPHIC REFEREE.

No syntax errors found. CIF dictionary Interpreting this report

Datablock: mu230926b_kk_a

Bond precision:	C-C = 0.0059 A	Wavelength=0.71073	
Cell:	a=16.4270(18)	b=39.005(4)	c=7.4785(9)
	alpha=90	beta=90	gamma=90
Temperature:	100 K		

	Calculated	Reported
Volume	4791.7(9)	4791.7(9)
Space group	F d d 2	F d d 2
Hall group	F 2 -2d	F 2 -2d
Moiety formula	C15 H10 Cl N O	C15 H10 Cl N O
Sum formula	C15 H10 Cl N O	C15 H10 Cl N O
Mr	255.69	255.69
Dx,g cm-3	1.418	1.418
Z	16	16
Mu (mm-1)	0.303	0.303
F000	2112.0	2112.0
F000’	2115.15	
h,k,lmax	19,46,8	19,46,8
Nref	2126[1152]	1919
Tmin,Tmax	0.913,0.953	0.770,0.950
Tmin’	0.877	

Correction method= # Reported T Limits: Tmin=0.770 Tmax=0.950
AbsCorr = MULTI-SCAN

Data completeness= 1.67/0.90 Theta(max)= 25.021

R(reflections)= 0.0412(1826) wR2(reflections)= 0.1324(1919)

S = 1.132 Npar= 163

The following ALERTS were generated. Each ALERT has the format
test-name_ALERT_alert-type_alert-level.
Click on the hyperlinks for more details of the test.

Alert level A

PLAT029_ALERT_3_A _diffrn_measured_fraction_theta_full value Low . 0.924 Why?

Author Response: The completeness was reduced because reflections from an additional crystal overlapped with low-angle reflections.

Alert level C

ABSTY02_ALERT_1_C An _exptl_absorpt_correction_type has been given without a literature citation. This should be contained in the _exptl_absorpt_process_details field.
Absorption correction given as Multi-Scan

PLAT089_ALERT_3_C Poor Data / Parameter Ratio (Zmax < 18) .... 7.07 Note

PLAT340_ALERT_3_C Low Bond Precision on C-C Bonds .... 0.00593 Ang.

Alert level G

PLAT012_ALERT_1_G N.O.K. _shelx_res_checksum Found in CIF ...... Please Check

PLAT883_ALERT_1_G Absent Datum for _atom_sites_solution_primary .. Please Do !

PLAT933_ALERT_2_G Number of HKL-OMIT Records in Embedded .res File 4 Note
1 5 1, 3 1 -1, 1 5 -1, 3 1 1,

1 **ALERT level A** = Most likely a serious problem - resolve or explain
0 **ALERT level B** = A potentially serious problem, consider carefully
3 **ALERT level C** = Check. Ensure it is not caused by an omission or oversight
3 **ALERT level G** = General information/check it is not something unexpected

3 ALERT type 1 CIF construction/syntax error, inconsistent or missing data
1 ALERT type 2 Indicator that the structure model may be wrong or deficient
3 ALERT type 3 Indicator that the structure quality may be low
0 ALERT type 4 Improvement, methodology, query or suggestion
0 ALERT type 5 Informative message, check

It is advisable to attempt to resolve as many as possible of the alerts in all categories. Often the minor alerts point to easily fixed oversights, errors and omissions in your CIF or refinement strategy, so attention to these fine details can be worthwhile. In order to resolve some of the more serious problems it may be necessary to carry out additional measurements or structure refinements. However, the purpose of your study may justify the reported deviations and the more serious of these should normally be commented upon in the discussion or experimental section of a paper or in the "special_details" fields of the CIF. checkCIF was carefully designed to identify outliers and unusual parameters, but every test has its limitations and alerts that are not important in a particular case may appear. Conversely, the absence of alerts does not guarantee there are no aspects of the results needing attention. It is up to the individual to critically assess their own results and, if necessary, seek expert advice.

Publication of your CIF in IUCr journals

A basic structural check has been run on your CIF. These basic checks will be run on all CIFs submitted for publication in IUCr journals (*Acta Crystallographica*, *Journal of Applied Crystallography*, *Journal of Synchrotron Radiation*); however, if you intend to submit to *Acta Crystallographica Section C* or *E* or *IUCrData*, you should make sure that full publication checks are run on the final version of your CIF prior to submission.

Publication of your CIF in other journals

Please refer to the *Notes for Authors* of the relevant journal for any special instructions relating to CIF submission.

PLATON version of 02/02/2025; check.def file version of 02/02/2025

Datablock mu230926b_kk_a - ellipsoid plot

ORTEP drawing of ***rac*-1b** showing thermal ellipsoids at the 50% probability level.

Single colorless crystals of ***rac*-1b** were obtained by vapour diffusion of hexane into a methanol solution of the compound at room temperature.

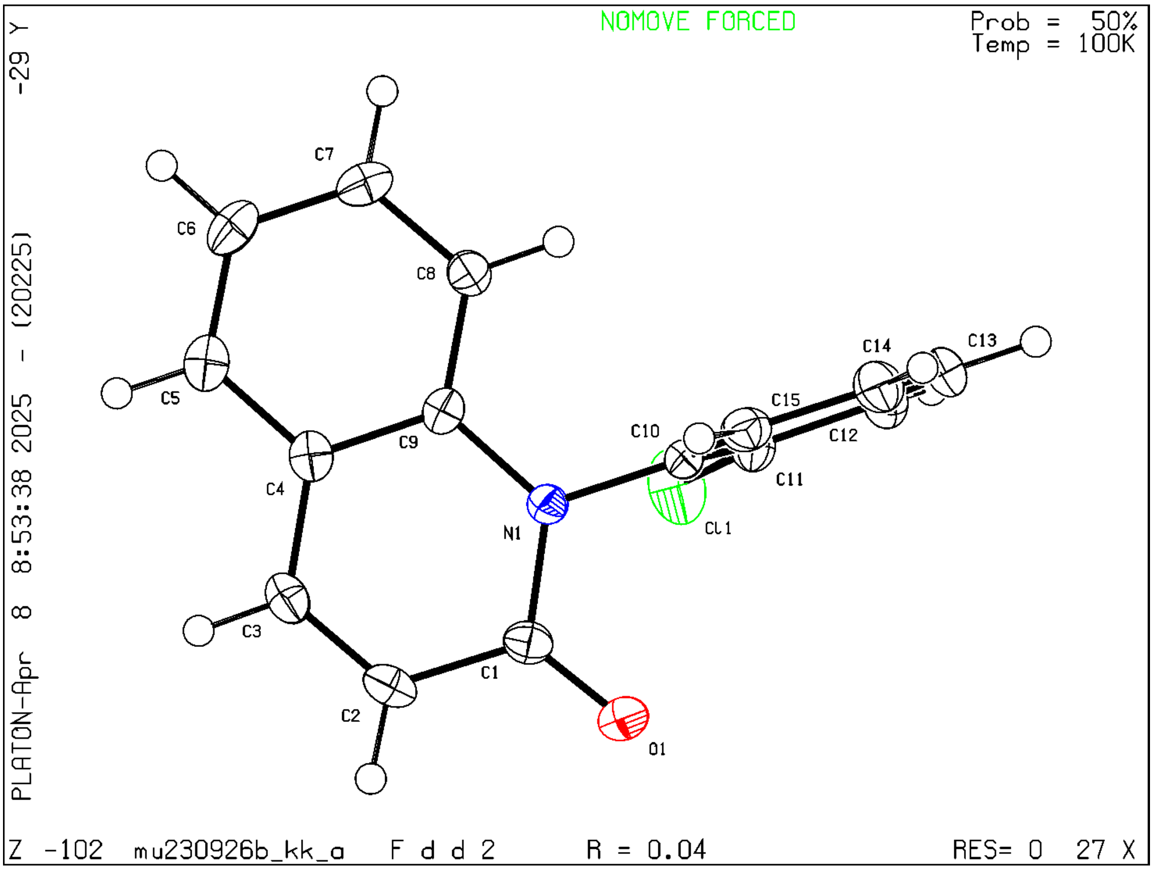

checkCIF/PLATON report

(*P*)-1b

Structure factors have been supplied for datablock(s) mu230926a3_a

THIS REPORT IS FOR GUIDANCE ONLY. IF USED AS PART OF A REVIEW PROCEDURE FOR PUBLICATION, IT SHOULD NOT REPLACE THE EXPERTISE OF AN EXPERIENCED CRYSTALLOGRAPHIC REFEREE.

No syntax errors found. CIF dictionary Interpreting this report

Datablock: mu230926a3_a

Bond precision:	C-C = 0.0030 A	Wavelength=0.71073	
Cell:	a=7.1125(11)	b=7.7261(16)	c=22.738(4)
	alpha=90	beta=90	gamma=90
Temperature:	100 K		

	Calculated	Reported
Volume	1249.5(4)	1249.5(4)
Space group	P 21 21 21	P 21 21 21
Hall group	P 2ac 2ab	P 2ac 2ab
Moiety formula	C15 H10 Cl N O	C15 H10 Cl N O
Sum formula	C15 H10 Cl N O	C15 H10 Cl N O
Mr	255.69	255.69
Dx,g cm-3	1.359	1.359
Z	4	4
Mu (mm-1)	0.291	0.291
F000	528.0	528.0
F000’	528.79	
h,k,lmax	8,9,27	8,9,27
Nref	2214[1315]	2205
Tmin,Tmax	0.907,0.926	0.790,0.930
Tmin’	0.879	

Correction method= # Reported T Limits: Tmin=0.790 Tmax=0.930
AbsCorr = MULTI-SCAN

Data completeness= 1.68/1.00 Theta(max)= 25.015

R(reflections)= 0.0283(2186) wR2(reflections)= 0.0734(2205)

S = 1.074 Npar= 163

The following ALERTS were generated. Each ALERT has the format
test-name_ALERT_alert-type_alert-level.
Click on the hyperlinks for more details of the test.

Alert level C

PLAT094_ALERT_2_C Ratio of Maximum / Minimum Residual Density .... 2.54 Report
PLAT911_ALERT_3_C Missing FCF Refl Between Thmin & STh/L= 0.595 3 Report
0 2 0, 1 0 2, 1 1 4,

Alert level G

PLAT033_ALERT_4_G Flack x Value Deviates > 3.0 * Sigma from Zero . 0.150 Note
PLAT883_ALERT_1_G Absent Datum for _atom_sites_solution_primary .. Please Do !
PLAT899_ALERT_4_G SHELXL2018 is Outdated and Succeeded by SHELXL 2019/3 Note
PLAT909_ALERT_3_G Percentage of I>2sig(I) Data at Theta(Max) Still 97% Note
PLAT910_ALERT_3_G Missing # of FCF Reflection(s) Below Theta(Min). 1 Note
0 0 2,
PLAT913_ALERT_3_G Missing # of Very Strong Reflections in FCF .... 2 Note
0 2 0, 1 0 2,
PLAT955_ALERT_1_G Reported (CIF) and Actual (FCF) Lmax Differ by . 1 Units
PLAT969_ALERT_5_G The 'Henn et al.' R-Factor-gap value .... 1.923 Note
Predicted wR2: Based on SigI**2 3.82 or SHELX Weight 6.83
PLAT978_ALERT_2_G Number C-C Bonds with Positive Residual Density. 3 Info

0 **ALERT level A** = Most likely a serious problem - resolve or explain
0 **ALERT level B** = A potentially serious problem, consider carefully
2 **ALERT level C** = Check. Ensure it is not caused by an omission or oversight
9 **ALERT level G** = General information/check it is not something unexpected

2 ALERT type 1 CIF construction/syntax error, inconsistent or missing data
2 ALERT type 2 Indicator that the structure model may be wrong or deficient
4 ALERT type 3 Indicator that the structure quality may be low
2 ALERT type 4 Improvement, methodology, query or suggestion
1 ALERT type 5 Informative message, check

It is advisable to attempt to resolve as many as possible of the alerts in all categories. Often the minor alerts point to easily fixed oversights, errors and omissions in your CIF or refinement strategy, so attention to these fine details can be worthwhile. In order to resolve some of the more serious problems it may be necessary to carry out additional measurements or structure refinements. However, the purpose of your study may justify the reported deviations and the more serious of these should normally be commented upon in the discussion or experimental section of a paper or in the "special_details" fields of the CIF. checkCIF was carefully designed to identify outliers and unusual parameters, but every test has its limitations and alerts that are not important in a particular case may appear. Conversely, the absence of alerts does not guarantee there are no aspects of the results needing attention. It is up to the individual to critically assess their own results and, if necessary, seek expert advice.

Publication of your CIF in IUCr journals

A basic structural check has been run on your CIF. These basic checks will be run on all CIFs submitted for publication in IUCr journals (*Acta Crystallographica*, *Journal of Applied Crystallography*, *Journal of Synchrotron Radiation*); however, if you intend to submit to *Acta Crystallographica Section C* or *E* or *IUCrData*, you should make sure that full publication checks are run on the final version of your CIF prior to submission.

Publication of your CIF in other journals

Please refer to the *Notes for Authors* of the relevant journal for any special instructions relating to CIF submission.

PLATON version of 02/02/2025; check.def file version of 02/02/2025

Datablock mu230926a3_a - ellipsoid plot

ORTEP drawing of **(*P*)-1b** showing thermal ellipsoids at the 50% probability level.

Single colorless crystals of **(*P*)-1b** were obtained by vapour diffusion of hexane into a methanol solution of the compound at room temperature.

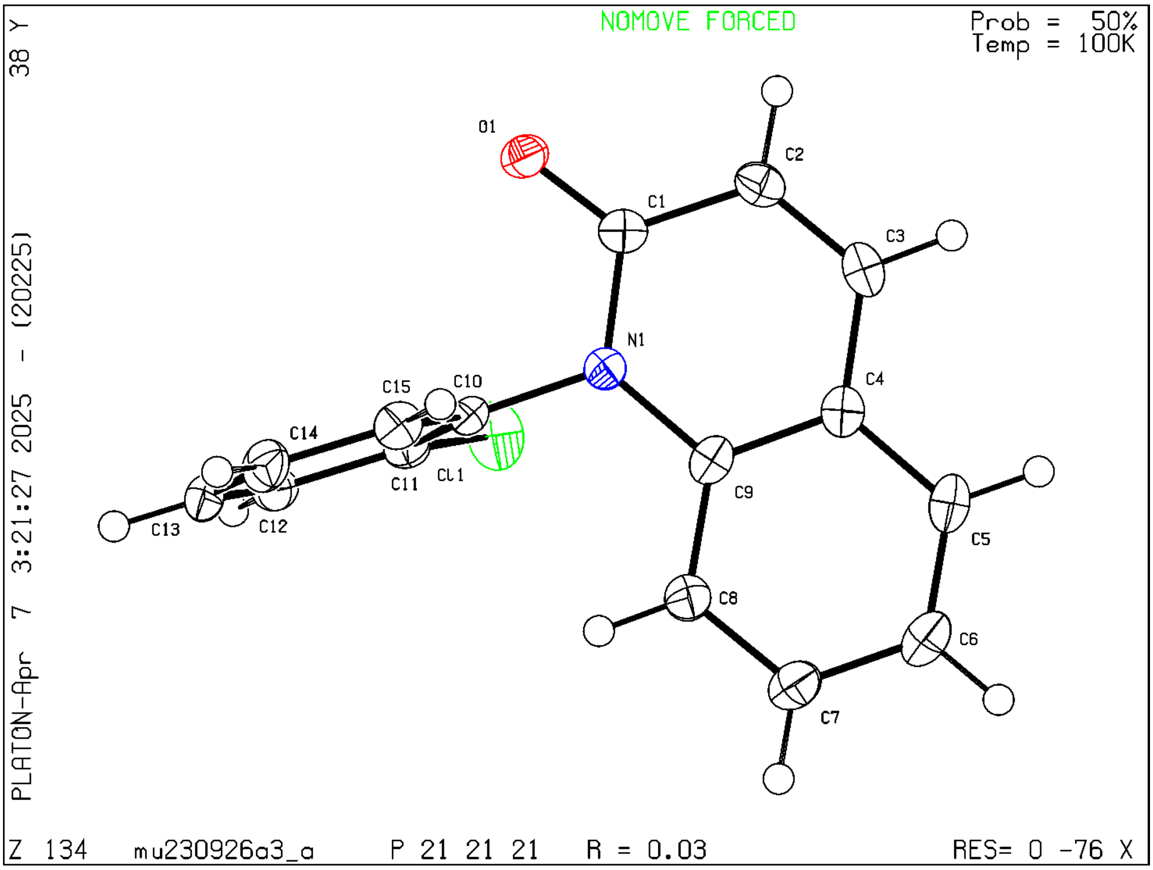

checkCIF/PLATON report

rac-2a

Structure factors have been supplied for datablock(s) MU240702b2_1_a

THIS REPORT IS FOR GUIDANCE ONLY. IF USED AS PART OF A REVIEW PROCEDURE FOR PUBLICATION, IT SHOULD NOT REPLACE THE EXPERTISE OF AN EXPERIENCED CRYSTALLOGRAPHIC REFEREE.

No syntax errors found. CIF dictionary Interpreting this report

Datablock: MU240702b2_1_a

Bond precision:	C-C = 0.0041 A		Wavelength=0.71073
Cell:	a=13.516(4)	b=12.303(4)	c=15.757(5)
	alpha=90	beta=90	gamma=90
Temperature:	100 K		

	Calculated	Reported
Volume	2620.2(14)	2620.1(13)
Space group	P b c a	P b c a
Hall group	-P 2ac 2ab	-P 2ac 2ab
Moiety formula	C15 H10 Br N S	C15 H10 Br N S
Sum formula	C15 H10 Br N S	C15 H10 Br N S
Mr	316.20	316.21
Dx,g cm-3	1.603	1.603
Z	8	8
Mu (mm-1)	3.276	3.276
F000	1264.0	1264.0
F000’	1263.09	
h,k,lmax	16,14,18	16,14,18
Nref	2322	2317
Tmin,Tmax	0.471,0.584	0.530,0.620
Tmin’	0.462	

Correction method= # Reported T Limits: Tmin=0.530 Tmax=0.620
AbsCorr = MULTI-SCAN

Data completeness= 0.998 Theta(max)= 25.023

R(reflections)= 0.0334(2019) wR2(reflections)= 0.0901(2317)

S = 1.052 Npar= 163

The following ALERTS were generated. Each ALERT has the format
test-name_ALERT_alert-type_alert-level.
Click on the hyperlinks for more details of the test.

Alert level C

PLAT911_ALERT_3_C Missing FCF Refl Between Thmin & STh/L= 0.595 5 Report
4 0 0, 0 0 2, 6 13 5, 5 12 9, 7 10 11,

Alert level G

PLAT883_ALERT_1_G Absent Datum for _atom_sites_solution_primary .. Please Do !
PLAT899_ALERT_4_G SHELXL2018 is Outdated and Succeeded by SHELXL 2019/3 Note
PLAT909_ALERT_3_G Percentage of I>2sig(I) Data at Theta(Max) Still 74% Note
PLAT913_ALERT_3_G Missing # of Very Strong Reflections in FCF .... 1 Note
4 0 0,
PLAT969_ALERT_5_G The 'Henn et al.' R-Factor-gap value .... 1.835 Note
Predicted wR2: Based on SigI**2 4.91 or SHELX Weight 8.56
PLAT978_ALERT_2_G Number C-C Bonds with Positive Residual Density. 3 Info

0 **ALERT level A** = Most likely a serious problem - resolve or explain
0 **ALERT level B** = A potentially serious problem, consider carefully
1 **ALERT level C** = Check. Ensure it is not caused by an omission or oversight
6 **ALERT level G** = General information/check it is not something unexpected

1 ALERT type 1 CIF construction/syntax error, inconsistent or missing data
1 ALERT type 2 Indicator that the structure model may be wrong or deficient
3 ALERT type 3 Indicator that the structure quality may be low
1 ALERT type 4 Improvement, methodology, query or suggestion
1 ALERT type 5 Informative message, check

It is advisable to attempt to resolve as many as possible of the alerts in all categories. Often the minor alerts point to easily fixed oversights, errors and omissions in your CIF or refinement strategy, so attention to these fine details can be worthwhile. In order to resolve some of the more serious problems it may be necessary to carry out additional measurements or structure refinements. However, the purpose of your study may justify the reported deviations and the more serious of these should normally be commented upon in the discussion or experimental section of a paper or in the "special_details" fields of the CIF. checkCIF was carefully designed to identify outliers and unusual parameters, but every test has its limitations and alerts that are not important in a particular case may appear. Conversely, the absence of alerts does not guarantee there are no aspects of the results needing attention. It is up to the individual to critically assess their own results and, if necessary, seek expert advice.

Publication of your CIF in IUCr journals

A basic structural check has been run on your CIF. These basic checks will be run on all CIFs submitted for publication in IUCr journals (*Acta Crystallographica*, *Journal of Applied Crystallography*, *Journal of Synchrotron Radiation*); however, if you intend to submit to *Acta Crystallographica Section C* or *E* or *IUCrData*, you should make sure that full publication checks are run on the final version of your CIF prior to submission.

Publication of your CIF in other journals

Please refer to the *Notes for Authors* of the relevant journal for any special instructions relating to CIF submission.

PLATON version of 02/02/2025; check.def file version of 02/02/2025

Datablock MU240702b2_1_a - ellipsoid plot

ORTEP drawing of ***rac*-2a** showing thermal ellipsoids at the 50% probability level.

Single yellow crystals of ***rac*-2a** were obtained from slow evaporation of hexane- methanol (1:1) mixture at room temperature.

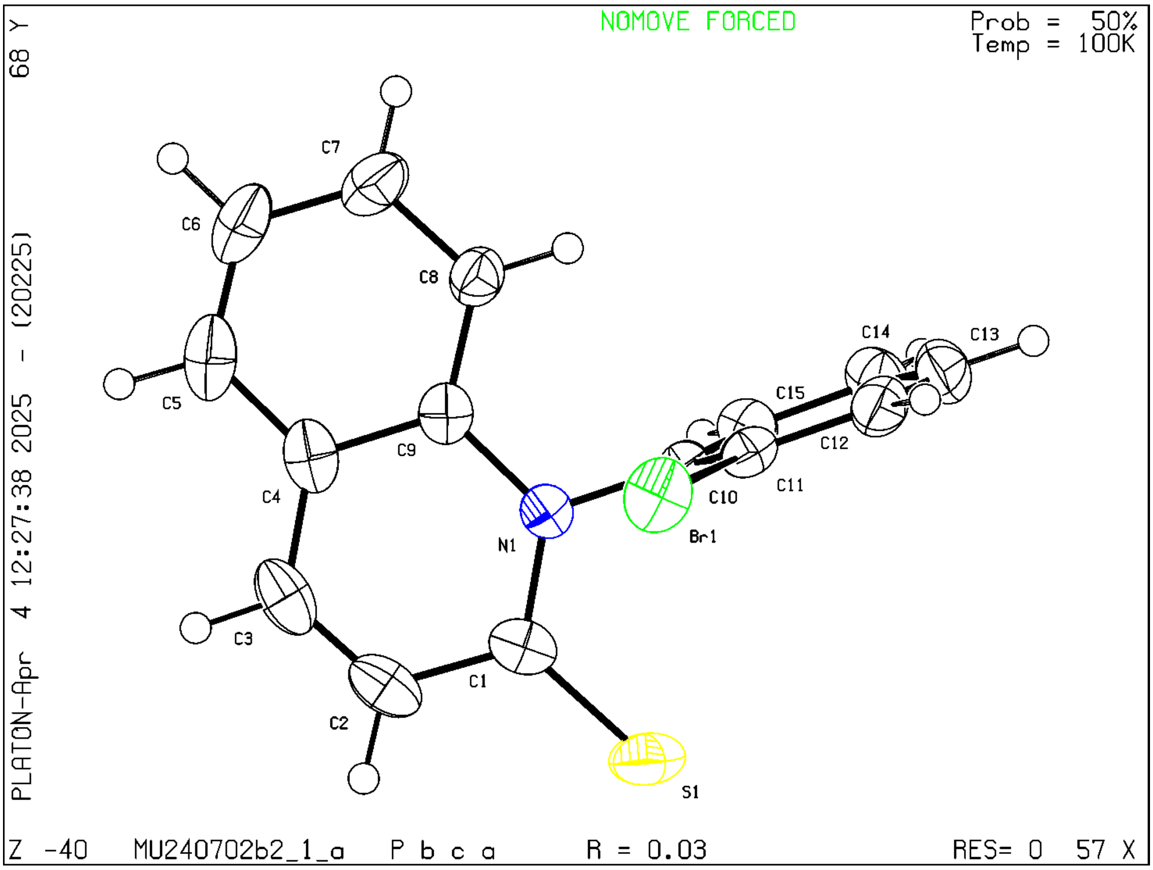

Supplement: File 1 — Experimental procedures for synthesis of compounds 1–4 and their spectral data, copies of 1H and 13C{1H} NMR charts of compounds 1–4, chiral MPLC and HPLC chart in compounds 1a,b, 2a, evaluation of rotational barriers of compounds 1a,b, and X-ray crystal data of rac-1a,b, (P)-1a,b, rac-2a (check CIF). [file Beilstein_J_Org_Chem-21-1748-s001.pdf]
